# Factors Associated with Changes in Capability-Wellbeing for Children and Young People of Secondary School Age During the First COVID-19 Lockdown

**DOI:** 10.1007/s12187-025-10294-y

**Published:** 2025-11-04

**Authors:** Isabella Floredin, Samantha Husbands, Jessica Hancock, Paul M. Mitchell, Joanna Coast, Emma Frew, Peymane Adab, Marie Murphy, Joanne Clarke, Miranda Pallan, Tim J. Peters

**Affiliations:** 1 Health Economics & Health Policy (HEHP@Bristol), Population Health Sciences, Bristol Medical School, University of Bristol, 1-5 Whiteladies Road, Bristol, BS8 1NU UK; 2 Health Economics Unit, Department of Applied Health Sciences, IOEM Building, University of Birmingham, Edgbaston, Birmingham, B15 2TT UK; 3 Department of Applied Health Sciences, Murray Learning Centre, University of Birmingham, Edgbaston, Birmingham, B15 2TT UK; 4 Bristol Dental School, University of Bristol, 1 Trinity Quay, Avon Street, Bristol, BS2 0PT UK

**Keywords:** Capability approach, Capability-wellbeing outcomes, Children and young people, COVID-19, Logistic regression

## Abstract

Concerns about the effects of the COVID-19 lockdown restrictions on children’s and young people’s wellbeing have been reported, but information about changes in wellbeing in terms of capabilities is limited. This study explores factors associated with changes in capability-wellbeing of children and young people during the first UK lockdown in March 2020. Data from an online survey of 687 participants aged 11–15 years was analysed using a staged logistic regression approach. Associations were explored between 25 potential explanatory variables across four groups (i. sociodemographic characteristics; ii. schooling and learning; iii. living situation; iv. other activities) and seven capability-wellbeing outcomes (safety and security; talking and support; having fun; achievement; relationships with: people I live with; family I don’t live with; friends). Odds ratios, 95% confidence intervals and p-values were used to identify independent associations. Several variables were associated with changes in capability-wellbeing from approximately 85% of the survey sample. Negative changes were associated with older age, girls, white-British ethnicity and owning no or one vehicle. Deprivation was associated with both negative and positive effects. Activities associated with negative change were more time watching TV and no time spent reading, learning new skills, playing games, and relaxing with household. Time spent talking to friends on social media was associated with both positive and negative changes. This study provides the first analysis of factors associated with changes in capability-wellbeing for children and young people during the first UK COVID-19 lockdown, identifying groups that may benefit from additional support in similar future contexts.

**Supplementary Information:**

The online version contains supplementary material available at 10.1007/s12187-025-10294-y.

## Introduction

The first COVID-19 lockdown (hereafter referred to as Lockdown 1) introduced on 23 March 2020 across the United Kingdom (UK) implemented social distancing restrictions (Institute for Government analysis, 2022b), bringing many changes to the lives of the whole population. For children and young people (CYP), daily activities such as physically attending school and other learning and social activities were disrupted for several months (Institute for Government analysis, 2022b). In particular, there were school closures over a fourteen-week period for all but vulnerable children and those of key workers (Department for Education, 2023; Timmins, 2021). Moreover, life at home, such as daily behaviour and social interactions, also changed.

Social distancing restrictions reduced COVID-19 virus transmission, and the associated demands on health care services, morbidity, and mortality (Hale et al., 2021; The Royal Society, 2023). Restrictions also resulted in adverse effects on wellbeing, with experiences differing across the population and CYP being particularly vulnerable to changes brought about by the restrictions (Imran et al., 2020). The effects of restrictions on CYP have been well-reported internationally, including studies focusing on physical activity, body weight, sleeping and sedentary behaviours (Kharel et al., 2022), mental health (Kauhanen et al., 2023; Marques de Miranda et al., 2020; Shoshani, 2023; Viner et al., 2022) and health-related quality of life (HRQoL) (Ravens-Sieberer et al., 2022) in early adolescence, a key developmental period for CYP.

Recommendations on mitigating the effects of restrictions on HRQoL, physical and mental health of CYP include the introduction of clinical interventions (Shoshani, 2023) and the implementation of health promotion and prevention strategies (Kharel et al., 2022; Ravens-Sieberer et al., 2022). Such recommendations are valuable for planning and implementing interventions and policies related to the health promotion of CYP. Yet, there have been shifts in health and social care policy making towards a more inclusive understanding of wellbeing, beyond health and HRQoL, in the UK and other high-income countries. The recent restructuring (2022) of decision-making bodies in the National Health Service (NHS) and implementation of Integrated Care Systems (ICSs) in England (Charles, 2022) is an example of such a shift. ICSs aim to deliver new models of integrated health and social care which places a greater emphasis on capturing the wider impact of interventions and addressing the social determinants of health in decision making (Murray & McKenna, 2020). Other countries, such as Canada, New Zealand, Iceland and Finland have also taken a similar approach with the introduction of policies focusing on broader wellbeing (Wellbeing Economy Alliance, 2021).

A focus on people’s capabilities is suggested to provide a broader evaluative framework (Coast et al., 2008; Verkerk et al., 2001) where the focus in not limited to health functionings but on the broader aspects of life that are important to people (that is their capabilities – the ‘beings and doings’) and to their wellbeing (Nussbaum & Sen, 1993). Capabilities focus on what a person is able to be and do in life, rather than what they actually do, regardless of whether they choose to do this (Nussbaum & Sen, 1993). The capability approach, initially developed by Amartya Sen (Sen, 1985, 1992) and Martha Nussbaum (Nussbaum, 2000, 2011), provides a normative and theoretical framework with a focus on the opportunities and freedom that people have to pursue the beings and doings that are important to them (Nussbaum & Sen, 1993). The capability approach has been widely used across many disciplines, including human development, public policy and healthcare (Robeyns, 2017), and can be a particularly useful framework in exploring the effects of the restrictions on people’s wellbeing and freedom to pursue important capabilities.

It is suggested that restrictions had a marked effect on people’s capabilities (Mitchell et al., 2023) but findings of this type have only been explored in adults to date (Himmler et al., 2023; Mitchell et al., 2023; Simon et al., 2021). The CONTRAST study survey aimed to explore the effects of social restrictions on CYP of secondary school age and collected capability information from those aged 11 to 15 years in the UK during Lockdown 1 (Pallan et al., 2021). Information collected in the survey suggested that different aspects of capability-wellbeing were positively or negatively affected (Pallan et al., 2021). Overall, during Lockdown 1, CYP felt as or more: safe and secure, able to talk to and seek support from others and able to feel close to household members. On the other hand, CYP felt less able to: have fun, to achieve and feel close to other family and friends (Pallan et al., 2021).

It is expected that a number of factors may have influenced these changes in capability, such as sociodemographic characteristics, living situation and daily activities across CYP. The present study aims to explore potential factors associated with changes in capability-wellbeing of CYP during Lockdown 1. In turn, this may help identify CYP groups at higher risk of reduced levels of capability-wellbeing and provide insights regarding potential protective factors that enable CYP to maintain or enhance their levels of capability-wellbeing. The findings could therefore inform courses of action to mitigate the long-term effects of COVID-19 on CYP as well as the impact of any future restrictions in a similar context.

## Methods

### Design

This study used data on capability-wellbeing from an online cross-sectional questionnaire survey, self-completed by CYP in the UK (England, Wales and Scotland), aged 11 to 15 years. The survey asked 67 questions in total, collecting information about i. before and ii. during Lockdown 1 (that is, recalled time periods: i. before 23 March 2020 and ii. from 23 March to the end of this lockdown, with the survey open until 31 July 2020) on the following areas: individual, family and household, schooling and learning, eating, activities, wellbeing and capability.

### Sampling

Sampling of secondary school age children (2019–20 academic year) aged 11 to 15 years and living in the UK employed a diverse range of methods. The survey and invitations to participate were circulated through existing research links with schools, young people’s networks (such as Girl Guides and Scouts Associations), professional and personal networks, and through social media (Facebook, Twitter, Instagram, Snapchat and TikTok) across the UK.

### Data Collection

The online survey was open for completion from 1 June to 31 July 2020, during which time there was limited reopening of schools (and non-essential retailers) in England and Wales (June) and the possibility of meeting other households indoors (plus some opening up of hospitality venues, hairdressers and places of worship) in England, Wales and Scotland (July). The full survey can be accessed through the CONTRAST webpage (www.birmingham.ac.uk/contrast-study). The online platform used for the survey was REDCap, which could be competed on any electronic device, including PC, laptop or smartphone.

Participants were required to complete an online consent form before accessing the survey. Ethical approval for the survey study was granted from [The University of Birmingham Research Ethics Committee (ERN_20-0645).].

The seven multiple-choice survey questions asked CYP participants how they feel at the moment compared with before lockdown in terms of different aspects of capability: security (*feeling safe and at ease)*; support *(talking and support from people who care about me)*; play and enjoyment (*having fun);* achievement (*being able to achieve things that are important to me);* attachment (*feeling close to…).* The question on attachment had three distinct parts: *feeling close to*: (i) *people I live with,* (ii) *family who I don’t live with,* (iii) *friends.* This distinction was made given the COVID-19 restrictions at the time on meeting other households. The five response options for all questions were: much more, more, no change, worse, much worse capability (the capability survey questions are given in full in Appendix 1).

Concepts included, and language used, in the survey capability questions drew from conceptual work to generate capability attributes for the development of new capability measures for CYP that was ongoing at the time (Husbands et al., 2024) and work from the existing ICECAP capability measure for adults (Al-Janabi et al., 2012).

### Data Analysis

Initial analysis described the completion of the seven capability questions, the distribution of responses and the extent to which responses across questions were similar or different, using two-way cross-tabulations between the outcome variables. Descriptive statistics assisted in determining whether (and if so, how) to dichotomise outcomes (for instance, negative change in capability versus no change or positive change in capability). A binary version of the outcomes was decided given the distribution of the outcomes, which in turn informed the nature of the regression approach used in the analysis (logistic regression), to explore factors associated with changes in capability-wellbeing of CYP during Lockdown 1. Logistic regression was directly applicable to the purpose of exploring associations with a number of factors, including estimates of magnitude – specifically, odds ratios and their 95% confidence intervals – and this was in accordance with our pre-specified analysis plan.

An exploratory staged logistic regression approach (Coast et al., 1996; Patel et al., 2005) was used to identify independent associations between the capability-wellbeing outcomes and possible explanatory variables. To generate binary outcomes, capability-wellbeing outcomes were dichotomised into the following categories: i) *‘worse or much worse capability’* (the two response options were combined and coded as one); ii) ‘*no change, more or much more capability’* (the three response options were combined and coded as zero). Although frequencies reported for each response option differed across the capability questions, the same dichotomy was used for all seven capability-wellbeing outcomes for consistency, and to aid in the interpretation of results, given that concerns about lockdowns were around their potential negative impacts.

Data from 25 potential explanatory variables collected in the survey were used in the analyses. Explanatory variables were divided into four conceptually similar groups:

i) **Sociodemographic characteristics (six variables):** Indices of Multiple Deprivation (IMD) quintiles (explored both as a categorical and continuous variable), age group, gender, ethnicity, and two proxies for socioeconomic status (SES) – specifically, whether the household has a dishwasher and number of vehicles owned by the household.
ii) **Schooling and learning (seven variables):** Time spent on schoolwork set by parents, hours of schoolwork set by school, time spent on live lessons per day, school type (state comprehensive school, state grammar school and private school), private academic tuition, private music and drama tuition, and whether CYP had their own computer for schoolwork.
iii) **Living situation (four variables):** House type, who looked after CYP in lockdown, outdoor space, and whether CYP had their own bedroom during Lockdown 1.
iv) **Other activities (including physical activity) (eight variables):** Time spent playing games per day, time spent relaxing with household per day, time spent exercising per day, number of times of doing exercise per week, time spent reading for fun per day, time spent talking to friends on social media per day, time spent watching TV per day, and time spent doing activities/skills per day.

The grouping of factors associated with this form of staged regression enabled insights to be generated separately for sets of conceptually related factors. It also ameliorated the potential problems of multicollinearity across the large number of variables.

As there were seven capability-wellbeing outcomes, seven separate analyses were conducted, one for each outcome. The first stage of the regression analysis developed univariable (unadjusted) models for each explanatory variable in turn, to determine whether that variable was associated with each capability-wellbeing outcome. To minimise the chances of omitting variables that might have a stronger association after adjusting for other factors (‘augmentation’ or ‘negative confounding’) at this first stage only variables with a p-value greater than or equal to 0.20 were excluded from further analysis.

The next stage of the regression analysis was to determine the statistically dominant factors within each of the four groups separately, by conducting multivariable (adjusted) analyses. For this (within-group) stage, the variables within each group were adjusted for every other variable in that group. A threshold of *p* < 0.10 was used to retain variables in these multivariable models and variables not meeting this threshold were dropped (no more than one at a time, to avoid missing instances of mutual confounding). Within-group models were re-run until all remaining variables within each group met the threshold level. Where only one variable remained within a group, the univariable p-value determined whether the variable met the 10% threshold level. For all multivariable analyses, IMD Quintile was included as a continuous variable unless there was a clearly non-linear pattern, in which case the categorical version was used; in any case both are presented in the univariable tables for completeness and to aid interpretation.

The remaining factors from within each group were then analysed with those of the other groups to produce a final set of factors that are independently associated with the capability-wellbeing outcome. Groups were combined sequentially in the above order for this ‘across-group’ multivariable regression (that is, variables from groups i. and ii., then i., ii. and iii and finally all four groups). For the across-group analyses, a threshold of *p* < 0.05 was used; each variable not meeting this criterion was dropped (again one at a time) and the model re-run. When all remaining variables met the 5% p-value threshold, variables from the next group were added. Although the primary model selection criterion throughout was based on p-values, at all stages in the analysis due consideration was paid to the magnitudes of the estimates and their confidence intervals, in particular when presenting the final model of factors independently associated with the outcome.

Analyses were conducted in STATA version 18, and at all stages the p-values presented have not been adjusted for multiple testing. In any event, these analyses should be considered exploratory, and emphasis will be placed on patterns of associations including their individual magnitudes (odds ratios and confidence intervals). ‘Prefer not to say’ responses were coded as missing for each variable and removed from the relevant analyses.

## Results

### Overview

Data were collected from 687 CYP in total in the CONTRAST survey. Participants were: 94% from England, 5% from Scotland and 1% from Wales. About half (53%) of the participants were female and 80% were of white ethnicity. The mean age of participants was 13.8 years and 81% attended a state-funded school. Detailed information on participant characteristics is available in the CONTRAST study report Appendix 1 (Pallan et al., 2021).

The distribution of responses across the seven capability questions are presented in Table 1, which demonstrates that aspects of CYP capabilities were both positively and negatively affected during Lockdown 1 compared with beforehand. With the outcome of worse/much worse versus no change/better/much better, for each capability in turn Supplementary Tables 1–7 present the results of the univariable logistic regression analyses for each of the 25 explanatory variables considered, with p-values under the 0.2 threshold depicted in bold to indicate which variables were taken forward to multivariable modelling. For the seven outcomes listed in Table 1, the number of explanatory variables consequently included in the multivariable modelling was 4, 7, 12, 12, 11, 5 and 7 respectively.

**Table 1:** Distributions of responses to the seven capability questions (outcomes)

Capability	Much more or more	No change	Worse or much worse	Total
Feeling safe and at ease	140 (24%)	360 (60%)	94 (16%)	594
Talking and support from people who care about me	132 (22%)	384 (65%)	78 (13%)	594
Having fun	159 (27%)	79 (13%)	357 (60%)	595
Achieve the things that are important to me	131 (22%)	136 (23%)	327(55%)	594
Relationships with people I live with	240 (41%)	299 (50%)	54 (9%)	593
Relationships with family I don’t live with	72 (12%)	343 (58%)	177 (30%)	592
Relationships with friends	100 (17%)	249 (42%)	243 (41%)	592

Tables 2, 3, 4, 5, 6, 7 and 8 present the results from the final multivariable logistic regression analyses. In the same order as above, the number of independent associations for the seven outcomes was 2, 1, 5, 4, 4, 2 and 2 respectively. Table 9 presents a summary of the results for the explanatory variables included in the univariable and final models for each of the seven outcomes. Of these 20 associations, while all met the minimum *p* < 0.05 threshold, eight indicated stronger evidence for these independent relationships (four at each of *p* < 0.01 and *p* < 0.001).

**Table 2:** Odds ratios, 95% confidence intervals and adjusted p-values from the final logistic regression model for the capability of feeling safe and at ease (odds ratios: ‘worse or much worse capability’ coded as one; ‘no change, more or much more capability’ coded as zero)

Explanatory variable	Reference category	Odds ratio	95% confidence interval	*p-*value
*Sociodemographic characteristics*				
Gender	Female			**0.0407**
Male		0.61	0.39, 0.98	
Own vehicle	0–1 vehicle			**0.0086**
2 or more vehicles		0.54	0.34, 0.86

**Table 3:** Odds ratios, 95% confidence intervals and adjusted p-values from the final logistic regression model for the capability of talking and support (odds ratios: ‘worse or much worse capability’ coded as one; ‘no change, more or much more capability’ coded as zero)

Explanatory variable	Reference category	Odds ratio	95% confidence interval	*p-*value
*Sociodemographic characteristics*				
Age group	11–12			**0.0476**
13		1.06	0.49, 2.28	
14		1.92	0.97, 3.79	
15–16		2.28	1.13, 4.57

**Table 4:** Odds ratios, 95% confidence intervals and adjusted p-values from the final logistic regression model for the capability to have fun (odds ratios: ‘worse or much worse capability’ coded as one; ‘no change, more or much more capability’ coded as zero

Explanatory variable	Reference category	Odds ratio	95% confidence interval	*p-*value
*Sociodemographic characteristics*				
IMD Quintile (continuous)		1.12	1.02, 1.36	**0.0232**
Ethnicity	White British			**0.0180**
Asian		0.53	0.27, 1.05	
Black		0.61	0.24, 1.56	
Mixed		0.85	0.37, 1.98	
White other		4.73	1.54, 14.54	
Other ethnic group		0.43	0.10, 1.92	
*Other activities*				
Time spent reading for fun per day	No time			**0.0035**
Up to 30 min		0.74	0.46, 1.18	
30 min—1 h		0.57	0.34, 0.98	
> 1 h		0.33	0.18, 0.60	
Time spent watching TV per day	No time			**0.0121**
30 min—1 h		2.50	1.26, 4.98	
1–3 h		3.20	1.67, 6.12	
4–6 h		2.40	1.12, 5.15	
> 6 h		2.08	0.89, 4.87	
Time spent playing games per day	No time			**0.0006**
Up to 30 min		0.46	0.26, 0.80	
30 min—1 h		0.34	0.20, 0.60	
1–3 h		0.47	0.27, 0.84	
> 3 h		0.23	0.15, 0.54

**Table 5:** Odds ratios, 95% confidence intervals and adjusted p-values from the final logistic regression model for the capability of achieving the things that are important to me (odds ratios: ‘worse or much worse capability’ coded as one; ‘no change, more or much more capability’ coded as zero)

Explanatory variable	Reference category	Odds ratio	95% confidence interval	*p-*value
*Sociodemographic characteristics*				
Gender	Female			**0.0016**
Male		0.56	0.39, 0.80	
*Other activities*				
Time spent reading for fun per day	No time			**0.0003**
Up to 30 min		0.70	0.45, 1.11	
30 min—1 h		0.65	0.39, 1.10	
> 1 h		0.25	0.13, 0.47	
Time spent watching TV per day	No time			**0.0129**
30 min—1 h		2.52	1.27, 5.00	
1–3 h		2.78	1.46, 5.27	
4–6 h		2.30	1.07, 4.91	
> 6 h		4.10	1.75, 9.58	
Time spent doing activities/skills per day	No time			**0.0031**
Up to 30 min		1.28	0.60, 2.72	
30 min—1 h		0.65	0.32, 1.33	
1–3 h		0.71	0.35, 1.46	
> 3 h		0.26	0.10, 0.72

**Table 6:** Odds ratios, 95% confidence intervals and adjusted p-values from the final logistic regression model for the capability to have relationships with people I live with (odds ratios: ‘worse or much worse capability’ coded as one; ‘no change, more or much more capability’ coded as zero)

Explanatory variable	Reference category	Odds ratio	95% confidence interval	*p*-value
*Sociodemographic characteristics*				
IMD Quintile (continuous)		0.75	0.60, 0.95	**0.0158**
*Other activities*				
Time spent talking to friends on social media per day	No time			**0.0349**
Up to 30 min		0.76	0.18, 3.22	
30 min—1 h		2.94	0.78, 11.06	
1–3 h		2.12	0.55, 8.17	
> 3 h		3.64	0.81, 16.41	
Time spent doing activities/skills at home	No time			**0.0312**
Up to 30 min		0.17	0.05, 0.53	
30 min—1 h		0.44	0.17, 1.12	
1–3 h		0.38	0.14, 1.01	
> 3 h		0.17	0.03, 0.92	
Time spent relaxing with household per day	No time			**0.0004**
Up to 30 min		0.37	0.14, 0.98	
30 min—1 h		0.16	0.06, 0.45	
1–3 h		0.10	0.03, 0.30	
> 3 h		0.15	0.04, 0.62

**Table 7:** Odds ratios, 95% confidence intervals and adjusted p-values from the final logistic regression model for the capability to have relationships with family I don’t live with (odds ratios: ‘worse or much worse capability’ coded as one; ‘no change, more or much more capability’ coded as zero)

Explanatory variable	Reference category	Odds ratio	95% confidence interval	*p-*value
*Other activities*				
Time spent playing games per day	No time			**0.0462**
Up to 30 min		0.70	0.41, 1.22	
30 min—1 h		0.67	0.39, 1.17	
1–3 h		0.62	0.35, 1.10	
> 3 h		1.41	0.80, 2.49	
Time spent relaxing with household per day	No time			**0.0292**
Up to 30 min		0.84	0.39, 1.81	
30 min—1 h		0.61	0.29, 1.31	
1–3 h		1.36	0.65, 2.86	
> 3 h		0.87	0.34, 2.21

**Table 8:** Odds ratios, 95% confidence intervals and adjusted *p*-values from the final logistic regression model for the capability to have relationships with friends (odds ratios: ‘worse or much worse capability’ coded as one; ‘no change, more or much more capability’ coded as zero)

Explanatory variable	Reference category	Odds ratio	95% confidence interval	*p*-value
*Sociodemographic characteristics*				
Ethnicity	White British			**0.0453**
Asian		0.70	0.37, 1.34	
Black		0.30	0.10, 0.94	
Mixed		0.64	0.28, 1.46	
White other		1.94	0.86, 4.39	
Other ethnic group		0.21	0.02, 1.73	
*Other activities*				
Time spent talking to friends on social media per day	No time			**0.0007**
Up to 30 min		0.79	0.42, 1.48	
30 min—1 h		0.54	0.29, 1.00	
1–3 h		0.37	0.20, 0.69	
> 3 h		0.29	0.13, 0.66

**Table 9:** Summary of *p*-values for explanatory variables taken forward to multivariable modelling (*p*-value threshold for inclusion < 0.20 at the univariable modelling stage) and variables retained in final model (*p*-value threshold for inclusion < 0.05 at the across-group modelling stage)

Explanatory variable	Feeling safe and at ease		Talking and support		Having fun		Achieve the things that are important to me		Relationships with people I live with		Relationships with family I don’t live with		Relationships with friends	
	Univar *p*-value	Final model *p*-value	Univar *p*-value	Final model *p*-value	Univar *p*-value	Final model *p*-value	Univar *p*-value	Final model *p*-value	Univar *p*-value	Final model *p*-value	Univar *p*-value	Final model *p*-value	Univar *p*-value	Final model *p*-value
*Sociodemographic characteristics*														
IMD Quintile (categorical)					0.0437		0.1735		0.0247		0.1910			
IMD Quintile (continuous)					0.0017	**0.0232**	0.0201		0.0143	**0.0158**			0.1243	
Age group (years)			0.0476	**0.0476**					0.0209					
Gender	0.0413	**0.0407**	0.0689				0.0244	**0.0016**						
Ethnicity					0.0093	**0.0180**							0.0418	**0.0453**
Own dishwasher					0.1530				0.1199					
Own vehicle	0.0102	**0.0086**	0.1881		0.1735				0.0892					
*Schooling and learning*														
Time spent on schoolwork set by parents			0.1102		0.1184		0.0617				0.0993			
Hours of schoolwork set by school									0.0982					
School type														
Time spent on live lessons per day														
Private academic tuition														
Private music and drama tuition													0.1717	
Own PC for schoolwork					0.1345									
*Living situation*														
House type							0.0398		0.1082					
Who looked after young person in lockdown	0.1231						0.0628		0.1656					
Outdoor space											0.0591			
Own bedroom in lockdown	0.1945				0.1498									
*Other activities*														
Time spent doing exercise per day							0.1477							
Number of times exercised per week			0.1561		0.1290		0.1356							
Time spent reading for fun per day					0.0118	**0.0035**	0.0001	**0.0003**						
Time spent talking to friends on social media per day									0.1880	**0.0349**			0.0008	**0.0007**
Time spent watching TV per day			0.1354		0.0076	**0.0121**	0.0046	**0.0129**	0.1200					
Time spent doing activities/skills per day					0.1180		0.0017	**0.0031**	0.0450	**0.0312**			0.1428	
Time spent playing games per day					0.0069	**0.0006**	0.0231				0.0471	**0.0462**	0.0595	
Time spent relaxing with household per day			0.1107				0.1480		0.0046	**0.0004**	0.0218	**0.0292**	0.1418	

For the purposes of describing these independent relationships across the seven outcomes, here we proceed from the perspective of the explanatory variables in their groups, again placing greater emphasis on patterns than isolated findings. (Details of a selection of the intermediate within-group and across-group models are given in Supplementary Tables 8–14, and due to constraints on space these are not discussed further here.)

### Associations with Capability-Wellbeing Outcomes for Sociodemographic Characteristics

During Lockdown 1 and compared with beforehand, some CYP groups were worse off than others in terms of different aspects of their capabilities in relation to sociodemographic characteristics. For instance, in terms of the increase in odds of a poor outcome (worse/much worse compared with before lockdown) for every unit increase in IMD quintile (from most to least deprived), there was a varying picture in respect of IMD. While CYP living in the less deprived areas were worse off in the odds of being able to do the things that they enjoy by 12% (*p* = 0.0232; Table 4), those in the more deprived areas were worse off in terms of the odds of being able to feel close to the people they live with by 33% (*p* = 0.0158; Table 6).

When comparing across the four age groups, overall, there was little or no evidence of differences in outcome for the seven capability-wellbeing outcomes. The only exception was weak evidence (*p* = 0.0476; Table 3) that older CYP were worse off than younger ones in terms of being able to talk to and seek support from people who cared about them (with the threshold appearing to be between 13 and 14 years of age), albeit that this was the only explanatory variable with even this level of evidence for an association with this outcome.

As shown in Tables 2 and 5 respectively, in terms of the odds of a poor outcome, girls were 64% worse off than boys in feeling safe and at ease (*p* = 0.0407) and 79% worse off in being able to achieve the things that were important to them (such as schoolwork, hobbies, interests and sports) (*p* = 0.0016).

In terms of ethnicity there were independent associations also for just two of the seven outcomes, and in neither case was the evidence strong (*p* = 0.018 and 0.045 for having fun and feeling close to friends respectively). As shown in Tables 4 and 8, for both these capability-wellbeing outcomes the pattern of adjusted odds ratios were very similar, with White British worse off than most other ethnicities (Asian, black, mixed and other); the exceptions of the CYP with the worst outcomes being those in the White other group had extremely wide confidence intervals (Tables 4 and 8) due to the small number in this category (*n* = 31, 4.5%) amongst the 687 respondents overall).

CYP from families owning no or one vehicle in the household were worse off in terms of their odds of feeling safe and at ease compared with those owning two or more vehicles by 85% (*p* = 0.0086; Table 2), but no such associations were observed in any of the other six final models.

### Associations with Capability-Wellbeing Outcomes for Schooling and Learning Variables, and for Living Situation Variables

While there were some variables relating to schooling and learning, and living situation variables, that passed the threshold for consideration in multivariable models, it is noteworthy that none of these associations were strong enough to be included in the final models after adjustment for variables in the other groups.

### Associations with Capability-Wellbeing Outcomes for Other Activities (Including physical activity)

It is also of note that of the 20 independent associations observed across the seven outcomes, 12 of them emanate from the group of variables relating to activities other than learning, including two of those with *p* < 0.01 and all four of those with the strongest evidence (*p* < 0.001). Highly consistent patterns are hard to discern – for instance, these 12 associations relate to two each across six of these eight characteristics (Table 9). There are some patterns, though, since almost all of these relationships are for just four outcomes: having fun, being able to achieve things and relationships with family the CYP do or don’t live with. Each of these 12 associations will now be described across three (potentially related) subsets of the six explanatory variables.

- Reading for fun, watching TV and talking to friends on social media
  CYP who did not spend time reading for fun and CYP who spent more time watching TV (including Netflix, YouTube and TikTok) were worse off in terms of being able to have fun (Table 4, *p*=0.0035 and 0.0121 respectively) and being able to achieve things important to them (Table 5, *p*=0.0003 and 0.0129 respectively). In terms of the nature and magnitudes of these associations, the odds of poor outcomes appeared to decline gradually as the time spent reading for fun increased, whereas there was more of a threshold effect for time watching TV with the odds of poor outcomes being increased however much time was involved (compared with no time spent watching TV (Tables 4 and 5).
  Talking to friends on social media (for example, Instagram, WhatsApp and Snapchat) had both a negative and positive effect on capabilities. CYP who spent 30 minutes or more talking to friends on social media were worse off than CYP who did not talk on social media in terms of feeling close to the people they live with; however, the evidence for this association was weak (Table 6; *p*= 0.0349) and the confidence intervals were wide. Much stronger evidence (Table 8; *p*=0.0007) was observed in terms of CYP’s ability to feel close to friends, with decreasing odds of poor outcomes on this capability as the time spent talking to friends on social media increased, down to below a third for those talking to friends on social media for four or more hours per day compared with CYP who spent no time at all on social media.
- Activities outside schoolwork to learn new knowledge and skills, including playing games
  CYP who spent less time doing activities outside of schoolwork to learn new knowledge and skills (for example, watching a TV documentary, home craft, cooking, learning a musical instrument) were worse off in terms of achievement and in relationships with family they lived with compared to those who spent more time on these activities, especially if this was more than 30 minutes per day (Table 5, *p*=0.0031 and Table 6, *p*=0.0312 respectively). For example, compared with those spending no time on such activities, those spending four or more hours per day were four to five times better off in terms of the odds of poor outcomes on these two capabilities (Tables 5 and 6).
  CYP who spent no time playing games (either alone or with friends; on a computer, Xbox, PlayStation, phone, Tablet, or other device) were worse off compared with those who were playing games in terms of being able to have fun (Table 4, *p*=0.0006) and being able to feel close to family outside the household (Table 7, *p*=0.0462). In both cases the main driver of the association was an increased odds (mostly between about two to four times) of a poor outcome amongst those who did not play games at all, and the apparent anomalous increased odds of a poor outcome in Table 7 for those spending four or more hours playing games may well be a chance finding.
- Relaxing with household members
  With one anomaly similar to the above, CYP who did not spend any time relaxing with household members were worse off compared with those who did in terms of feeling close to people they live with and close to family they did not live with (Table 6, *p*=0.0004 and Table 7, *p*=0.0292 respectively). The magnitude of these effects varied and were greater for people the CYP live with (up to 10 times for those spending over 30 minutes a day; Table 6) than for family they do not live with (a 15% to 30% benefit; Table 7).

## Discussion

This study explored factors associated with change in capability-wellbeing outcomes of CYP during the first UK COVID-19 lockdown in 2020 and compared with beforehand. A number of factors (*n* = 25) were explored using a staged approach to logistic regression analysis. Five sociodemographic and six activity-related factors were found to be associated with different capability-wellbeing outcomes. Sociodemographic and living situation factors associated with negative changes were older age (talking and support), being female (achieving, safety and security), being of white-British ethnicity (having fun, relationships with friends), and owning no or one vehicle in the household (safety and security). Higher deprivation (IMD) was associated with both negative (relationships with people I live with) and positive (having fun) changes. Activities associated with negative change were more time spent watching TV (achieving, having fun) and no time spent: reading for fun (achieving, having fun), doing activities to learn new knowledge and skills (achieving, relationships with people I live with), playing games (having fun, relationships with family I don’t live with) and relaxing with household (relationships with: people I live with, family I don’t live with). More time spent talking to friends on social media was associated with both positive (relationships with friends) and negative (relationships with people I live with) changes in capability-wellbeing.

Existing studies from high-, upper-middle and lower-middle-income countries with variations in health coverage, welfare systems and levels of pandemic-related restrictions highlight associations between sociodemographic factors and CYP’s physical and mental health in the context of the pandemic (Kauhanen et al., 2023; Kharel et al., 2022; Lorthe et al., 2023; Panchal et al., 2023; Scrimin et al., 2022; Viner et al., 2022). This study provides additional evidence of a differential impact across sociodemographic background on capability-wellbeing outcomes in the UK, which is a high-income country with universal health coverage and relatively high levels of restrictions implemented (Roser, 2021).

During the early pandemic, families in lower-paid jobs were more likely to be affected by economic uncertainty (Mirela Miescu, 2023) and health worries (Scrimin et al., 2022) internationally. Perhaps this uncertainty may have placed a strain on relationships between household members and CYP from more deprived areas evident in the present study findings. Moreover, households from more deprived areas tend to have a greater proportion of manual workers, such as supermarket staff, sanitation and waste management workers and delivery workers, who provided essential services during the pandemic (Dütsch, 2022). It is likely that these essential workers were less present at home during the pandemic, resulting in fewer opportunities to spend time with CYP in the household.

CYP with higher SES are more likely to take part in extra-curricular activities such as arts and sports, activities that provide opportunities to have fun (Michael Donnelly et al., 2019). Such activities, were particularly disrupted in Lockdown 1 due to the restrictions in place (Institute for Government analysis, 2022a). This may help explain the difference found between CYP from the least and most deprived areas for the outcome of being able to have fun.

It is suggested that the mental health of adolescent girls in the UK (Montero-Marin et al., 2023) and other countries, such as Australia (Magson et al., 2021), Iceland (Thorisdottir et al., 2021) and China (Chen et al., 2021) was more affected than that of boys during the pandemic. The findings on capability-wellbeing outcomes highlight particular aspects of mental wellbeing, around safety, security and achievement, which were adversely affected for girls early on in the pandemic. In contrast, a study in Germany suggested a higher increase in mental health problems among boys than girls, yet these findings were based on parental proxy rather than self-reports (Ravens-Sieberer et al., 2022).

Existing COVID-19 studies conducted in different countries, including Europe, China and Canada, suggest that older adolescents reported more negative emotions (Chen et al., 2020; Panchal et al., 2023; Ren et al., 2021) and stress related to more loneliness (Ellis et al., 2020) compared with younger CYP. This is consistent with the difference found between older and younger CYP for the talking and support outcome. These findings may be due to the different psychosocial developmental stage CYP are at with identity forming in older adolescents who seek connection through relationships with peers rather than family (Erikson, 1950).

Previous studies have explored associations between different activities and CYP’s mental health and wellbeing. It is suggested that screen-related sedentary behaviour has adverse effects on the cognitive, physical, emotional, and social development in adolescence (Brunetti et al., 2016; Camerini et al., 2022; Giedd, 2012). This is consistent with the negative associations found between watching TV and the achieving and having fun outcomes. Yet, talking to friends on social media, an activity involving more screen time, was positively associated with closeness to friends in this study. The positive effects of social interaction with friends during the pandemic on CYP’s mental wellbeing have been previously highlighted (Montero-Marin et al., 2023). Perhaps the more interactive, rather than passive, nature of this latter activity may help explain its positive relationship with capability-wellbeing.

The positive associations of reading (Clark & Picton, 2020) and playing (Dodd et al., 2023; James et al., 2021) with CYP’s mental wellbeing previously reported also align with those found in this study between: reading and the achieving and enjoyment outcomes, and doing activities outside schoolwork and closeness to household members.

Previous COVID-19 studies in France, Germany and the UK, highlight the negative effects on quality of life and mental health of CYP with limited living space (Bourion-Bédès et al., 2022; Ravens-Sieberer et al., 2022) and CYP in disadvantaged schools (Moss et al., 2020; Rebecca Montacute et al., 2022). While such associations were expected in this study, none of the variables explored in the schooling and learning and living situation groups were retained in any of the final models. Indeed, school type, private academic tuition and time spent on live lessons never got past the first stage of the analysis for any of the capability-wellbeing outcomes. It is possible that this difference in findings relates to the types of outcomes being measured with the set of capability-wellbeing questions used in this study capturing different aspects of quality of life and mental health.

Study data were collected in the UK and provide useful information on the factors that were associated with falls in capability-wellbeing during the Lockdown 1 restrictions on CYP in the UK. Data were collected during the lockdown period, close in time to the initial shock of restrictions being imposed, and from a relatively large sample.

There are, however, some limitations to the work. The survey was cross-sectional, and data are only available for Lockdown 1. A longitudinal survey, capturing the subsequent lockdowns and later stages of the pandemic may have highlighted greater variation in factors and outcomes associated with capability-wellbeing. In a similar vein, there were no pre-pandemic data available, so questions had to be framed in terms of changes relative to pre-lockdown, rather than being able to look directly at change from measures collected pre-pandemic and during Lockdown 1. While this retrospective, compared with ‘pre-’ and ‘post-’, approach to data collection is open to bias (Blome & Augustin, 2015), the approach was useful in this study (Lamb, 2005) and in a previous COVID-19 study on adult capability-wellbeing (Mitchell et al., 2023) given the lack of contemporaneous ‘pre- ‘ data on CYP’s capability-wellbeing.

Participants from a range of socioeconomic backgrounds were represented in the survey but the varied approach to recruitment meant that some groups were less represented than others. CYP from less affluent families were underrepresented in particular and while the ethnicity of participants was broadly similar to that of the overall English population, participants of mixed ethnicity were slightly underrepresented compared with the ethnic breakdown for the age group of 11–15 years (Office for National Statistics, 2023; Pallan et al., 2021).

The work was conducted at a time when interviews to explore important aspects of capability-wellbeing in this age group were underway, but neither data collection nor analysis were complete. The questions asked therefore drew on these early interviews and the ICECAP-A measure, which meant that some aspects of capability-wellbeing that were later found to be important to CYP of this age were not included in this study, particularly around the ability to learn and discover new things, and the ability to think positively about the future (Husbands et al., 2024).

Given the sample size available, the five response options for each capability question were grouped into two response options for the (logistic) regression analyses, with ‘no change’ in capability combined with ‘better or much better’ capability. Any interpretations of the analyses in terms of CYP groups reporting being better off must be treated with caution given that these groups include those CYP who reported ‘no change’ in capability. In the context of the overall negative impact of the pandemic and associated restrictions on wellbeing, however, it is valid to consider a ‘no change’ to capability as a positive rather than a negative outcome.

Although a staged approach to the regression analysis was used to address potential issues of multicollinearity, the study was exploratory in nature and findings can only provide insights into associations between the explanatory variables and capability-wellbeing outcomes rather than to identify causal relationships. Moreover, due to the large number of statistical tests conducted any interpretations on the variables remaining in the final models should be treated with caution and the width of some of the confidence intervals should also be considered.

Notwithstanding these limitations, this study provides insight into factors associated with changes in capability-wellbeing during the early stages of the COVID-19 pandemic and can be used both to inform policy and decision makers in their planning and support of CYP from this age group in the long-term, and to enhance planning for future contexts where similar social restrictions might need to be implemented. Findings can be used to identify potential risk groups, such as CYP with lower SES, who may benefit from additional support in the long-term and in similar future contexts. Findings can also inform decision makers about potential protective factors that may reduce the loss in CYP’s capability-wellbeing, such as social connection and doing meaningful learning activities outside schoolwork, that they might be able to promote and prioritise in such situations. Findings can also inform decision makers around risk factors, such as screen-related sedentary behaviour, which they might be able to discourage.

Although a number of overlaps between factors and outcomes are evident in the associations found, the findings suggest that different capabilities are associated with different explanatory variables. This observation reiterates the need to develop CYP’s capability-wellbeing outcome measures (Husbands et al., 2022, 2024; Mitchell et al., 2021) and capture change independently for all the different aspects of a person’s wellbeing.

Furthermore, the current study highlights the importance of including capability-wellbeing measures within longitudinal surveys along with measures of health status and subjective wellbeing so that a direct ‘pre-’ and ‘post-’ comparison of capability-wellbeing is possible. Given that capability-wellbeing measurement is increasingly acknowledged as being important in policy (Amuthavalli Thiyagarajan et al., 2022), the availability of ‘pre- ‘ and ‘post-’ capability-wellbeing data and relevant comparisons may help inform decision making on appropriate policies on the impact on CYP’s wellbeing during similar future contexts.

## Conclusions

The present study provided the first analysis of factors associated with CYP’s capability change during restrictions early on in the pandemic. While the impact of restrictions on physical and mental wellbeing and HRQoL has been previously explored, this study provides a fuller picture of the impact of restrictions on broader aspects of capability-wellbeing. To address inequalities and promote better wellbeing among CYP and their families, policies that consider CYP individual and family needs and vulnerabilities and policies that foster meaningful relationships and activities for CYP are needed.

## Electronic supplementary material

Below is the link to the electronic supplementary material.

Supplementary file1 (DOCX 183 KB)

## Data Availability

Anonymised study data and related materials are available from the study Chief Investigator, Miranda Pallan on reasonable request.

## Appendix 1

Survey capability questions:

For each question below, comparing before lockdown with now, please tick which statement best describes how you feel at the moment.

1. **Feeling safe and at ease**
  - I feel much more safe and at ease than I did before lockdown
  - I feel more safe and at ease than I did before lockdown
  - I feel as safe and at ease as I did before lockdown
  - I feel less safe and at ease than I did before lockdown
  - I feel much less safe and at ease than I did before lockdown
2. **Talking and support from people who care about me**
  - I am able to talk to and seek support from the people who are there for me, much more than I was before lockdown
  - I am able to talk to and seek support from the people who are there for me, more than I was before lockdown
  - I am able to talk to and seek support from the people who are there for me, as much as I was before lockdown
  - I am able to talk to and seek support from the people who are there for me, less than I was before lockdown
  - I am able to talk to and seek support from the people who are there for me, much less than I was before lockdown
3. **Having fun**
  - I am able to do a lot more of the things that I enjoy than I was before lockdown
  - I am able to do more of the things that I enjoy than I was before lockdown
  - I able to do as many of the things that I enjoy as I was before lockdown
  - I am able to do fewer of the things that I enjoy than I was before lockdown
  - I am able to do a lot fewer of the things that I enjoy than I was before lockdown
4. **Being able to achieve things that are important to me (these might be things like, schoolwork, hobbies & interests, and sports)**
  - I am able to achieve much more of what is important to me than I was before lockdown
  - I am able to achieve more of what is important to me than I was before lockdown
  - I am able to achieve as much of what is important to me as I was before lockdown
  - I am able to achieve less of what is important to me than I was before lockdown
  - I am able to achieve much less of what is important to me than I was before lockdown
  A. Relationships
5. **Relationships with people I live with**
  - My ability to feel close to the people I live with is much better than before lockdown
  - My ability to feel close to the people I live with is better than before lockdown
  - My ability to feel close to the people I live with is the same as before lockdown
  - My ability to feel close to the people I live with is worse than before lockdown
  - My ability to feel close to the people I live with is much worse than before lockdown
6. **Relationships with family who I don’t live with**
  - My ability to feel close to family who I don’t live with, is much better than before lockdown
  - My ability to feel close to family who I don’t live with, is better than before lockdown
  - My ability to feel close to family who I don’t live with, is the same as before lockdown
  - My ability to feel close to family who I don’t live with, is worse than before lockdown
  - My ability to feel close to family who I don’t live with, is much worse than before lockdown
7. **Relationships with friends**
  - My ability to feel close to friends is much better than before lockdown
  - My ability to feel close to friends is better than before lockdown
  - My ability to feel close to friends is the same as before lockdown
  - My ability to feel close to friends is worse than before lockdown
  - My ability to feel close to friends is much worse than before lockdown
