# Supplementary material for: Factors Associated with Changes in Capability-Wellbeing for Children and Young People of Secondary School Age During the First COVID-19 Lockdown
Source: Child Indic Res. 2025 Nov 4;19(1):87–111. doi: 10.1007/s12187-025-10294-y (PMC12876456; doi:10.1007/s12187-025-10294-y)
Supplement: Supplementary file 1 — Supplementary file1 (DOCX 183 KB) [file 12187_2025_10294_MOESM1_ESM.docx]

**SUPPLEMENTARY TABLES**

*Supplementary Table 1 - Univariable logistic regression analyses for the capability of feeling safe and at ease (odds ratios: ‘worse or much worse capability’ coded as one; ‘no change, more or much more capability’ coded as zero)*

| Explanatory variable | Reference category | Odds ratio^a^ | 95% confidence interval^a^ | p*-*value from univariable model^b^ |
| --- | --- | --- | --- | --- |
| *Sociodemographic characteristics* |  |  |  |  |
| IMD Quintile (categorical) | IMD 1 |  |  | 0.9338 |
| IMD 2 |  | 0.77 | 0.32, 1.85 |  |
| IMD 3 |  | 0.76 | 0.35, 1.67 |  |
| IMD 4 |  | 0.93 | 0.44, 1.99 |  |
| IMD 5 |  | 0.79 | 0.38, 1.62 |  |
|  |  |  |  |  |
| IMD Quintile (continuous) |  | 0.97 | 0.83, 1.15 | 0.7608 |
|  |  |  |  |  |
| Age group (years) | 11-12 |  |  | 0.5216 |
| 13 |  | 1.34 | 0.71, 2.50 |  |
| 14 |  | 1.40 | 0.76, 2.59 |  |
| 15-16 |  | 1.59 | 0.84, 3.02 |  |
|  |  |  |  |  |
| Gender | Female |  |  | **0.0413** |
| Male |  | 0.62 | 0.39, 0.98 |  |
|  |  |  |  |  |
| Ethnicity | White British |  |  | 0.7207 |
| Asian |  | 1.44 | 0.67, 3.13 |  |
| Black |  | 1.75 | 0.62, 4.90 |  |
| Mixed |  | 1.24 | 0.46, 3.36 |  |
| White other |  | 1.70 | 0.66, 4.36 |  |
| Other ethnic group |  | 0.85 | 0.10, 7.01 |  |
|  |  |  |  |  |
| Own dishwasher | No |  |  | 0.3763 |
| Yes |  | 0.80 | 0.48, 1.31 |  |
|  |  |  |  |  |
| Own vehicle | 0-1 vehicle |  |  | **0.0102** |
| 2 or more vehicles |  | 0.55 | 0.35, 0.87 |  |
|  |  |  |  |  |
| *Schooling and learning* |  |  |  |  |
| Time spent on schoolwork set by parents | No time |  |  | 0.7863 |
| Some time |  | 0.76 | 0.56, 1.55 |  |
|  |  |  |  |  |
| Hours of schoolwork set by school | Less than 1 hour |  |  | 0.7070 |
| 1-3 hours |  | 0.84 | 0.39, 1.79 |  |
| >3 hours |  | 0.75 | 0.36, 1.54 |  |
|  |  |  |  |  |
| School type | State comprehensive school |  |  | 0.6532 |
| State grammar school |  | 0.97 | 0.52, 1.79 |  |
| Private school |  | 1.27 | 0.74, 2.18 |  |
|  |  |  |  |  |
| Time spent on live lessons per day | No time |  |  | 0.2308 |
| Some time |  | 0.76 | 0.48, 1.19 |  |
|  |  |  |  |  |
| Private academic tuition | No |  |  | 0.8535 |
| Yes |  | 0.93 | 0.44, 1.96 |  |
|  |  |  |  |  |
| Private music and drama tuition | No |  |  | 0.6486 |
| Yes |  | 0.90 | 0.56, 1.44 |  |
|  |  |  |  |  |
| Own PC for schoolwork | Yes |  |  | **0.0282** |
| No |  | 1.67 | 1.06, 2.64 |  |
|  |  |  |  |  |
| *Living situation* |  |  |  |  |
| House type | Other type of home |  |  | 0.4080 |
| House |  | 0.67 | 0.27, 1.71 |  |
|  |  |  |  |  |
| Who looked after young person in lockdown | Mother and father |  |  | **0.1231** |
| Other |  | 1.47 | 0.90, 2.40 |  |
|  |  |  |  |  |
| Outdoor space | No access to outdoor space |  |  | 0.7960 |
| Private garden or outdoor space |  | 0.65 | 0.13, 3.17 |  |
| Shared garden or outdoor space |  | 0.82 | 0.12, 5.57 |  |
|  |  |  |  |  |
| Own bedroom in lockdown | No |  |  | **0.1945** |
| Yes |  | 0.66 | 0.36, 1.23 |  |
|  |  |  |  |  |
| *Other activities* |  |  |  |  |
| Time spent doing exercise per day | No time |  |  | 0.7093 |
| Up to 30 minutes |  | 1.12 | 0.42, 3.00 |  |
| 30 minutes - 1 hour |  | 1.15 | 0.45, 2.95 |  |
| 1-3 hours |  | 0.77 | 0.29, 2.06 |  |
| >3 hours |  | 1.07 | 0.29, 3.90 |  |
|  |  |  |  |  |
| Number of times exercised per week | Less than once per week |  |  | 0.5609 |
| 1-3 times |  | 0.84 | 0.43, 1.65 |  |
| 4-5 times |  | 0.67 | 0.32, 1.39 |  |
| >5 times |  | 0.64 | 0.31, 1.35 |  |
|  |  |  |  |  |
| Time spent reading for fun per day | No time |  |  | 0.4203 |
| Up to 30 minutes |  | 0.67 | 0.38, 1.15 |  |
| 30 minutes - 1 hour |  | 1.00 | 0.55, 1.84 |  |
| >1 hours |  | 0.76 | 0.36, 1.61 |  |
|  |  |  |  |  |
| Time spent talking to friends on social media per day | No time |  |  | 0.2003 |
| Up to 30 minutes |  | 0.45 | 0.20, 0.99 |  |
| 30 minutes - 1 hour |  | 0.84 | 0.40, 1.75 |  |
| 1-3 hours |  | 0.67 | 0.32, 1.41 |  |
| >3 hours |  | 0.46 | 0.16, 1.32 |  |
|  |  |  |  |  |
| Time spent watching TV per day | No time |  |  | 0.3486 |
| 30 minutes - 1 hour |  | 0.96 | 0.37, 2.48 |  |
| 1-3 hours |  | 1.55 | 0.66, 3.64 |  |
| 4-6 hours |  | 1.14 | 0.41, 3.14 |  |
| >6 hours |  | 1.94 | 0.70, 5.38 |  |
|  |  |  |  |  |
| Time spent doing activities/skills per day | No time |  |  | 0.2327 |
| Up to 30 minutes |  | 1.36 | 0.52, 3.59 |  |
| 30 minutes - 1 hour |  | 1.52 | 0.60, 3.84 |  |
| 1-3 hours |  | 0.99 | 0.37, 2.60 |  |
| >3 hours |  | 2.625 | 0.83, 8.28 |  |
|  |  |  |  |  |
| Time spent playing games per day | No time |  |  | 0.8153 |
| Up to 30 minutes |  | 0.87 | 0.44, 1.73 |  |
| 30 minutes - 1 hour |  | 1.30 | 0.68, 2.48 |  |
| 1-3 hours |  | 1.15 | 0.59, 2.25 |  |
| >3 hours |  | 0.98 | 0.46, 2.08 |  |
|  |  |  |  |  |
| Time spent relaxing with household per day | No time |  |  | 0.2150 |
| Up to 30 minutes |  | 1.04 | 0.44, 2.49 |  |
| 30 minutes - 1 hour |  | 0.52 | 0.21, 1.28 |  |
| 1-3 hours |  | 0.74 | 0.31, 1.79 |  |
| >3 hours |  | 0.61 | 0.19, 1.95 |  |

^a^ Odds ratios and their 95% confidence intervals portray the direction and nature of the association with the capability-wellbeing outcome.
^b^ Univariable Wald tests; variables with p-values <0.2 were taken forward to within-group modelling stages.

*Supplementary Table 2 - Univariable logistic regression analyses for the capability of talking and support (odds ratios: ‘worse or much worse capability’ coded as one; ‘no change, more or much more capability’ coded as zero)*

| Explanatory variable | Reference category | Odds ratio^a^ | 95% confidence interval^a^ | p*-*value from univariable model^b^ |
| --- | --- | --- | --- | --- |
| *Sociodemographic characteristics* |  |  |  |  |
| IMD Quintile (categorical) | IMD 1 |  |  | 0.9356 |
| IMD 2 |  | 1.17 | 0.44, 3.17 |  |
| IMD 3 |  | 0.99 | 0.39, 2.50 |  |
| IMD 4 |  | 1.19 | 0.49, 2.93 |  |
| IMD 5 |  | 1.31 | 0.57, 3.04 |  |
|  |  |  |  |  |
| IMD Quintile (continuous) |  | 1.07 | 0.89, 1.28 | 0.4746 |
|  |  |  |  |  |
| Age group (years) | 11-12 |  |  | **0.0476** |
| 13 |  | 1.06 | 0.49, 2.28 |  |
| 14 |  | 1.92 | 0.97, 3.79 |  |
| 15-16 |  | 2.28 | 1.13, 4.57 |  |
|  |  |  |  |  |
| Gender | Female |  |  | **0.0689** |
| Male |  | 0.63 | 0.38, 1.04 |  |
|  |  |  |  |  |
| Ethnicity | White British |  |  | 0.8845 |
| Asian |  | 0.83 | 0.32, 2.19 |  |
| Black |  | 0.66 | 0.15, 2.92 |  |
| Mixed |  | 1.06 | 0.36, 3.16 |  |
| White other |  | 1.51 | 0.55, 4.14 |  |
| Other ethnic group |  | 1 | - |  |
|  |  |  |  |  |
| Own dishwasher | No |  |  | 0.7414 |
| Yes |  | 0.91 | 0.52, 1.59 |  |
|  |  |  |  |  |
| Own vehicle | 0-1 vehicle |  |  | **0.1881** |
| 2 or more vehicles |  | 1.44 | 0.84, 2.47 |  |
|  |  |  |  |  |
| *Schooling and learning* |  |  |  |  |
| Time spent on schoolwork set by parents | No time |  |  | **0.1102** |
| Some time |  | 0.61 | 0.34, 1.12 |  |
|  |  |  |  |  |
| Hours of schoolwork set by school | Less than 1 hour |  |  | 0.6279 |
| 1-3 hours |  | 0.69 | 0.31, 1.54 |  |
| >3 hours |  | 0.70 | 0.33, 1.49 |  |
|  |  |  |  |  |
| School type | State comprehensive school |  |  | 0.2567 |
| State grammar school |  | 0.53 | 0.24, 1.16 |  |
| Private School |  | 1.04 | 0.57, 1.88 |  |
|  |  |  |  |  |
| Time spent on live lessons per day | No time |  |  | 0.5280 |
| Some time |  | 0.85 | 0.52, 1.39 |  |
|  |  |  |  |  |
| Private academic tuition | No |  |  | 0.7233 |
| Yes |  | 0.86 | 0.38, 1.97 |  |
|  |  |  |  |  |
| Private music and drama tuition | No |  |  | 0.3717 |
| Yes |  | 0.79 | 0.47, 1.33 |  |
|  |  |  |  |  |
| Own PC for schoolwork | Yes |  |  | 0.3371 |
| No |  | 1.28 | 0.77, 2.12 |  |
|  |  |  |  |  |
| *Living situation* |  |  |  |  |
| House type | Other type of home |  |  | 0.8531 |
| House |  | 0.90 | 0.30, 2.67 |  |
|  |  |  |  |  |
| Who looked after young person in lockdown | Mother and father |  |  | 0.4785 |
| Other |  | 1.22 | 0.71, 2.09 |  |
|  |  |  |  |  |
| Outdoor space | No access to outdoor space |  |  | 0.6484 |
| Private garden or outdoor space |  | 0.53 | 0.11, 2.59 |  |
| Shared garden or outdoor space |  | 0.37 | 0.04, 3.14 |  |
|  |  |  |  |  |
| Own bedroom in lockdown | No |  |  | 0.9037 |
| Yes |  | 1.05 | 0.50, 2.20 |  |
|  |  |  |  |  |
| *Other activities* |  |  |  |  |
| Time spent doing exercise per day | No time |  |  | 0.3876 |
| Up to 30 minutes |  | 0.71 | 0.29, 1.76 |  |
| 30 minutes - 1 hour |  | 0.45 | 0.18, 1.10 |  |
| 1-3 hours |  | 0.51 | 0.21, 1.27 |  |
| >3 hours |  | 0.58 | 0.15, 2.14 |  |
|  |  |  |  |  |
| Number of times exercised per week | Less than once per week |  |  | **0.1561** |
| 1-3 times |  | 0.53 | 0.27, 1.04 |  |
| 4-5 times |  | 0.43 | 0.20, 0.94 |  |
| >5 times |  | 0.52 | 0.25, 1.08 |  |
|  |  |  |  |  |
| Time spent reading for fun per day | No time |  |  | 0.3109 |
| Up to 30 minutes |  | 0.70 | 0.38, 1.28 |  |
| 30 minutes - 1 hour |  | 0.92 | 0.46, 1.81 |  |
| >1 hours |  | 1.37 | 0.66, 2.82 |  |
|  |  |  |  |  |
| Time spent talking to friends on social media per day | No time |  |  | 0.6869 |
| Up to 30 minutes |  | 1.60 | 0.57, 4.49 |  |
| 30 minutes - 1 hour |  | 1.80 | 0.65, 5.01 |  |
| 1-3 hours |  | 1.45 | 0.52, 4.03 |  |
| >3 hours |  | 2.23 | 0.69, 7.15 |  |
|  |  |  |  |  |
| Time spent watching TV per day | No time |  |  | **0.1354** |
| 30 minutes - 1 hour |  | 0.49 | 0.18, 1.31 |  |
| 1-3 hours |  | 0.90 | 0.39, 2.07 |  |
| 4-6 hours |  | 1.28 | 0.50, 3.29 |  |
| >6 hours |  | 1.50 | 0.55, 4.06 |  |
|  |  |  |  |  |
| Time spent doing activities/skills per day | No time |  |  | 0.3153 |
| Up to 30 minutes |  | 0.95 | 0.39, 2.32 |  |
| 30 minutes - 1 hour |  | 0.50 | 0.20, 1.23 |  |
| 1-3 hours |  | 0.88 | 0.37, 2.10 |  |
| >3 hours |  | 0.91 | 0.27, 3.09 |  |
|  |  |  |  |  |
| Time spent playing games per day | No time |  |  | 0.6389 |
| Up to 30 minutes |  | 0.76 | 0.36, 1.62 |  |
| 30 minutes - 1 hour |  | 0.93 | 0.45, 1.95 |  |
| 1-3 hours |  | 0.91 | 0.43, 1.91 |  |
| >3 hours |  | 1.42 | 0.67, 2.99 |  |
|  |  |  |  |  |
| Time spent relaxing with household per day | No time |  |  | **0.1107** |
| Up to 30 minutes |  | 0.54 | 0.23, 1.26 |  |
| 30 minutes - 1 hour |  | 0.41 | 0.18, 0.96 |  |
| 1-3 hours |  | 0.33 | 0.14, 0.81 |  |
| >3 hours |  | 0.29 | 0.08, 1.02 |  |

^a^ Odds ratios and their 95% confidence intervals portray the direction and nature of the association with the capability-wellbeing outcome.
^b^ Univariable Wald tests; variables with p-values <0.2 were taken forward to within-group modelling stages.

*Supplementary Table 3 - Univariable logistic regression analyses for the capability to have fun (odds ratios: ‘worse or much worse capability’ coded as one; ‘no change, more or much more capability’ coded as zero)*

| Explanatory variable | Reference category | Odds ratio^a^ | 95% confidence interval^a^ | p*-*value from univariable model^b^ |
| --- | --- | --- | --- | --- |
| *Sociodemographic characteristics* |  |  |  |  |
| IMD Quintile (categorical) | IMD 1 |  |  | **0.0437** |
| IMD 2 |  | 1.03 | 0.54, 1.95 |  |
| IMD 3 |  | 1.40 | 0.78, 2.51 |  |
| IMD 4 |  | 1.63 | 0.91, 2.92 |  |
| IMD 5 |  | 2.05 | 1.18, 3.56 |  |
|  |  |  |  |  |
| IMD Quintile (continuous) |  | 1.21 | 1.07, 1.37 | **0.0017** |
|  |  |  |  |  |
| Age group (years) | 11-12 |  |  | 0.8725 |
| 13 |  | 0.99 | 0.63, 1.55 |  |
| 14 |  | 1.16 | 0.74, 1.82 |  |
| 15-16 |  | 0.96 | 0.60, 1.55 |  |
|  |  |  |  |  |
| Gender | Female |  |  | 0.5483 |
| Male |  | 0.90 | 0.65, 1.26 |  |
|  |  |  |  |  |
| Ethnicity | White British |  |  | **0.0093** |
| Asian |  | 0.48 | 0.26, 0.89 |  |
| Black |  | 0.52 | 0.22, 1.24 |  |
| Mixed |  | 0.77 | 0.36, 1.64 |  |
| White other |  | 3.61 | 1.23, 10.61 |  |
| Other ethnic group |  | 0.38 | 0.09, 1.60 |  |
|  |  |  |  |  |
| Own dishwasher | No |  |  | **0.1530** |
| Yes |  | 1.32 | 0.90, 1.95 |  |
|  |  |  |  |  |
| Own vehicle | 0-1 vehicle |  |  | **0.1735** |
| 2 or more vehicles |  | 1.27 | 0.90, 1.81 |  |
|  |  |  |  |  |
| *Schooling and learning* |  |  |  |  |
| Time spent on schoolwork set by parents | No time |  |  | **0.1184** |
| Some time |  | 0.75 | 0.52, 1.08 |  |
|  |  |  |  |  |
| Hours of schoolwork set by school | Less than 1 hour |  |  | 0.3635 |
| 1-3 hours |  | 1.15 | 0.64, 2.05 |  |
| >3 hours |  | 1.39 | 0.80, 2.44 |  |
|  |  |  |  |  |
| School type | State comprehensive school |  |  | 0.4771 |
| State grammar school |  | 0.87 | 0.56, 1.35 |  |
| Private school |  | 1.21 | 0.79, 1.86 |  |
|  |  |  |  |  |
| Time spent on live lessons per day | No time |  |  | 0.9830 |
| Some time |  | 1.00 | 0.72, 1.40 |  |
|  |  |  |  |  |
| Private academic tuition | No |  |  | 0.4052 |
| Yes |  | 0.80 | 0.47, 1.36 |  |
|  |  |  |  |  |
| Private music and drama tuition | No |  |  | 0.9520 |
| Yes |  | 0.99 | 0.70, 1.40 |  |
|  |  |  |  |  |
| Own PC for schoolwork | Yes |  |  | **0.1345** |
| No |  | 1.32 | 0.92, 1.90 |  |
|  |  |  |  |  |
| *Living situation* |  |  |  |  |
| House type | Other type of home |  |  | 0.2716 |
| House |  | 1.53 | 0.72, 3.27 |  |
|  |  |  |  |  |
| Who looked after young person in lockdown | Mother and father |  |  | 0.8888 |
| Other |  | 0.90 | 0.62, 1.32 |  |
|  |  |  |  |  |
| Outdoor space | No access to outdoor space |  |  | 0.2957 |
| Private garden or outdoor space |  | 0.43 | 0.09, 2.10 |  |
| Shared garden or outdoor space |  | 0.26 | 0.04, 1.56 |  |
|  |  |  |  |  |
| Own bedroom in lockdown | No |  |  | **0.1498** |
| Yes |  | 1.44 | 0.88, 2.37 |  |
|  |  |  |  |  |
| *Other activities* |  |  |  |  |
| Time spent doing exercise per day | No time |  |  | 0.8184 |
| Up to 30 minutes |  | 0.95 | 0.46, 1.99 |  |
| 30 minutes - 1 hour |  | 1.11 | 0.55, 2.26 |  |
| 1-3 hours |  | 0.92 | 0.45, 1.89 |  |
| >3 hours |  | 0.74 | 0.28, 1.96 |  |
|  |  |  |  |  |
| Number of times exercised per week | Less than once per week |  |  | **0.1290** |
| 1-3 times |  | 0.93 | 0.55, 1.57 |  |
| 4-5 times |  | 1.57 | 0.88, 2.79 |  |
| >5 times |  | 1.05 | 0.60, 1.82 |  |
|  |  |  |  |  |
| Time spent reading for fun per day | No time |  |  | **0.0118** |
| Up to 30 minutes |  | 0.78 | 0.51, 1.18 |  |
| 30 minutes - 1 hour |  | 0.64 | 0.39, 1.03 |  |
| >1 hours |  | 0.40 | 0.23, 0.70 |  |
|  |  |  |  |  |
| Time spent talking to friends on social media per day | No time |  |  | 0.7040 |
| Up to 30 minutes |  | 1.12 | 0.61, 2.07 |  |
| 30 minutes - 1 hour |  | 1.35 | 0.73, 2.49 |  |
| 1-3 hours |  | 1.41 | 0.77, 2.57 |  |
| >3 hours |  | 1.07 | 0.50, 2.29 |  |
|  |  |  |  |  |
| Time spent watching TV per day | No time |  |  | **0.0076** |
| 30 minutes - 1 hour |  | 2.34 | 1.24, 4.39 |  |
| 1-3 hours |  | 2.66 | 1.48, 4.77 |  |
| 4-6 hours |  | 1.90 | 0.96, 3.78 |  |
| >6 hours |  | 1.38 | 0.65, 2.89 |  |
|  |  |  |  |  |
| Time spent doing activities/skills per day | No time |  |  | **0.1180** |
| Up to 30 minutes |  | 1.31 | 0.66, 2.58 |  |
| 30 minutes - 1 hour |  | 0.91 | 0.48, 1.72 |  |
| 1-3 hours |  | 0.90 | 0.47, 1.73 |  |
| >3 hours |  | 0.47 | 0.19, 1.15 |  |
|  |  |  |  |  |
| Time spent playing games per day | No time |  |  | **0.0069** |
| Up to 30 minutes |  | 0.49 | 0.29, 0.83 |  |
| 30 minutes - 1 hour |  | 0.44 | 0.26, 0.75 |  |
| 1-3 hours |  | 0.58 | 0.34, 0.99 |  |
| >3 hours |  | 0.39 | 0.22, 0.68 |  |
|  |  |  |  |  |
| Time spent relaxing with household per day | No time |  |  | 0.3434 |
| Up to 30 minutes |  | 1.16 | 0.57, 2.38 |  |
| 30 minutes - 1 hour |  | 1.14 | 0.57, 2.31 |  |
| 1-3 hours |  | 0.88 | 0.43, 1.78 |  |
| >3 hours |  | 0.64 | 0.27, 1.51 |  |

^a^ Odds ratios and their 95% confidence intervals portray the direction and nature of the association with the capability-wellbeing outcome.
^b^ Univariable Wald tests: variables with p-values <0.2 were taken forward to within-group modelling stages.

*Supplementary Table 4 - Univariable logistic regression analyses for the capability of achieving the things that are important to me (odds ratios: ‘worse or much worse capability’ coded as one; ‘no change, more or much more capability’ coded as zero)*

| Explanatory variable | Reference category | Odds ratio^a^ | 95% confidence interval^a^ | p*-*value from univariable model^b^ |
| --- | --- | --- | --- | --- |
| *Sociodemographic characteristics* |  |  |  |  |
| IMD Quintile (categorical) | IMD 1 |  |  | **0.1735** |
| IMD 2 |  | 1.00 | 0.53, 1.91 |  |
| IMD 3 |  | 1.18 | 0.66, 2.11 |  |
| IMD 4 |  | 1.22 | 0.68, 2.16 |  |
| IMD 5 |  | 1.72 | 0.99, 2.97 |  |
|  |  |  |  |  |
| IMD Quintile (continuous) |  | 1.15 | 1.02, 1.30 | **0.0201** |
|  |  |  |  |  |
| Age group (years) | 11-12 |  |  | 0.2865 |
| 13 |  | 1.44 | 0.92, 2.26 |  |
| 14 |  | 0.94 | 0.60, 1.45 |  |
| 15-16 |  | 1.16 | 0.72, 1.86 |  |
|  |  |  |  |  |
| Gender | Female |  |  | **0.0244** |
| Male |  | 0.68 | 0.49, 0.95 |  |
|  |  |  |  |  |
| Ethnicity | White British |  |  | 0.4795 |
| Asian |  | 0.61 | 0.33, 1.13 |  |
| Black |  | 0.80 | 0.34, 1.87 |  |
| Mixed |  | 1.12 | 0.53, 2.41 |  |
| White other |  | 1.35 | 0.61, 3.02 |  |
| Other ethnic group |  | 0.48 | 0.11, 2.02 |  |
|  |  |  |  |  |
| Own dishwasher | No |  |  | 0.2093 |
| Yes |  | 1.28 | 0.87, 1.87 |  |
|  |  |  |  |  |
| Own vehicle | 0-1 vehicle |  |  | 0.7778 |
| 2 or more vehicles |  | 1.05 | 0.74, 1.49 |  |
|  |  |  |  |  |
| *Schooling and learning* |  |  |  |  |
| Time spent on schoolwork set by parents | No time |  |  | **0.0617** |
| Some time |  | 0.70 | 0.49, 1.02 |  |
|  |  |  |  |  |
| Hours of schoolwork set by school | Less than 1 hour |  |  | 0.8614 |
| 1-3 hours |  | 0.90 | 0.50, 1.61 |  |
| >3 hours |  | 0.99 | 0.56, 1.72 |  |
|  |  |  |  |  |
| School type | State comprehensive school |  |  | 0.3460 |
| State grammar school |  | 0.79 | 0.51, 1.23 |  |
| Private school |  | 1.18 | 0.78, 1.80 |  |
|  |  |  |  |  |
| Time spent on live lessons per day | No time |  |  | 0.9818 |
| Some time |  | 1.00 | 0.72, 1.39 |  |
|  |  |  |  |  |
| Private academic tuition | No |  |  | 0.7780 |
| Yes |  | 0.93 | 0.54, 1.58 |  |
|  |  |  |  |  |
| Private music and drama tuition | No |  |  | 0.5052 |
| Yes |  | 0.89 | 0.63, 1.25 |  |
|  |  |  |  |  |
| Own PC for schoolwork | Yes |  |  | 0.4704 |
| No |  | 1.14 | 0.80, 1.62 |  |
|  |  |  |  |  |
| *Living situation* |  |  |  |  |
| House type | Other type of home |  |  | **0.0398** |
| House |  | 2.29 | 1.04, 5.05 |  |
|  |  |  |  |  |
| Who looked after young person in lockdown | Mother and father |  |  | **0.0628** |
| Other |  | 0.70 | 0.48, 1.02 |  |
|  |  |  |  |  |
| Outdoor space | No access to outdoor space |  |  | 0.6333 |
| Private garden or outdoor space |  | 1.56 | 0.41, 5.87 |  |
| Shared garden or outdoor space |  | 1.14 | 0.24, 5.46 |  |
|  |  |  |  |  |
| Own bedroom in lockdown | No |  |  | 0.4332 |
| Yes |  | 1.21 | 0.74, 2.00 |  |
|  |  |  |  |  |
| *Other activities* |  |  |  |  |
| Time spent doing exercise per day | No time |  |  | **0.1477** |
| Up to 30 minutes |  | 0.87 | 0.41, 1.83 |  |
| 30 minutes - 1 hour |  | 0.79 | 0.39, 1.61 |  |
| 1-3 hours |  | 0.54 | 0.26, 1.12 |  |
| >3 hours |  | 0.51 | 0.19, 1.35 |  |
|  |  |  |  |  |
| Number of times exercised per week | Less than once per week |  |  | **0.1356** |
| 1-3 times |  | 1.14 | 0.67, 1.93 |  |
| 4-5 times |  | 1.31 | 0.75, 2.30 |  |
| >5 times |  | 0.78 | 0.45, 1.35 |  |
|  |  |  |  |  |
| Time spent reading for fun per day | No time |  |  | **0.0001** |
| Up to 30 minutes |  | 0.67 | 0.44, 1.01 |  |
| 30 minutes - 1 hour |  | 0.69 | 0.43, 1.12 |  |
| >1 hours |  | 0.26 | 0.14, 0.46 |  |
|  |  |  |  |  |
| Time spent talking to friends on social media per day | No time |  |  | 0.8563 |
| Up to 30 minutes |  | 0.95 | 0.52, 1.75 |  |
| 30 minutes - 1 hour |  | 0.96 | 0.52, 1.76 |  |
| 1-3 hours |  | 1.00 | 0.55, 1.82 |  |
| >3 hours |  | 1.36 | 0.63, 2.94 |  |
|  |  |  |  |  |
| Time spent watching TV per day | No time |  |  | **0.0046** |
| 30 minutes - 1 hour |  | 2.71 | 1.41, 5.21 |  |
| 1-3 hours |  | 3.09 | 1.67, 5.69 |  |
| 4-6 hours |  | 2.58 | 1.27, 5.24 |  |
| >6 hours |  | 3.9 | 1.78, 8.52 |  |
|  |  |  |  |  |
| Time spent doing activities/skills per day | No time |  |  | **0.0017** |
| Up to 30 minutes |  | 1.20 | 0.61, 2.38 |  |
| 30 minutes - 1 hour |  | 0.59 | 0.31, 1.13 |  |
| 1-3 hours |  | 0.66 | 0.34, 1.27 |  |
| >3 hours |  | 0.29 | 0.11, 0.73 |  |
|  |  |  |  |  |
| Time spent playing games per day | No time |  |  | **0.0231** |
| Up to 30 minutes |  | 0.57 | 0.35, 0.94 |  |
| 30 minutes - 1 hour |  | 0.50 | 0.30, 0.82 |  |
| 1-3 hours |  | 0.82 | 0.49, 1.37 |  |
| >3 hours |  | 0.50 | 0.29, 0.87 |  |
|  |  |  |  |  |
| Time spent relaxing with household per day | No time |  |  | **0.1480** |
| Up to 30 minutes |  | 0.81 | 0.40, 1.65 |  |
| 30 minutes - 1 hour |  | 0.95 | 0.47, 1.91 |  |
| 1-3 hours |  | 0.77 | 0.38, 1.55 |  |
| >3 hours |  | 0.40 | 0.17, 0.97 |  |

^a^ Odds ratios and their 95% confidence intervals portray the direction and nature of the association with the capability-wellbeing outcome.
^b^ Univariable Wald tests: variables with p-values <0.2 were taken forward to within-group modelling stages.

*Supplementary Table 5 - Univariable logistic regression analyses for the capability to have relationships with the people I live with (odds ratios: ‘worse or much worse capability’ coded as one; ‘no change, more or much more capability’ coded as zero)*

| Explanatory variable | Reference category | Odds ratio^a^ | 95% confidence interval^a^ | p-value from univariable model^b^ |
| --- | --- | --- | --- | --- |
| *Sociodemographic characteristics* |  |  |  |  |
| IMD Quintile  (categorical) | IMD 1 |  |  | **0.0247** |
| IMD 2 |  |  | 0.24, 1.48 |  |
| IMD 3 |  |  | 0.08, 6.27 |  |
| IMD 4 |  |  | 0.18, 0.99 |  |
| IMD 5 |  |  | 0.14, 0.75 |  |
|  |  |  |  |  |
| IMD Quintile  (continuous) |  | 0.78 | 0.63, 0.95 | **0.0143** |
|  |  |  |  |  |
| Age group (years) | 11-12 |  |  | **0.0209** |
| 13 |  | 1.48 | 0.58, 3.74 |  |
| 14 |  | 2.02 | 0.85, 4.81 |  |
| 15-16 |  | 3.51 | 1.52, 8.10 |  |
|  |  |  |  |  |
| Gender | Female |  |  | 0.2682 |
| Male |  | 0.72 | 0.40, 1.29 |  |
|  |  |  |  |  |
| Ethnicity | White British |  |  | 0.3421 |
| Asian |  | 0.46 | 0.11, 1.97 |  |
| Black |  | 0.48 | 0.06, 3.68 |  |
| Mixed |  | 1.17 | 0.34, 4.03 |  |
| White other |  | 2.31 | 0.83, 6.41 |  |
| Other ethnic group |  | 1.00 | .. |  |
|  |  |  |  |  |
| Own dishwasher | No |  |  | **0.1199** |
| Yes |  | 0.62 | 0.34, 1.13 |  |
|  |  |  |  |  |
| Own vehicle | 0-1 vehicle |  |  | **0.0892** |
| 2 or more vehicles |  | 0.61 | 0.35, 1.14 |  |
|  |  |  |  |  |
| *Schooling and learning* |  |  |  |  |
| Time spent on schoolwork set by parents | No time |  |  | 0.4306 |
| Some time |  | 1.28 | 0.70, 2.35 |  |
|  |  |  |  |  |
| Hours of schoolwork set by school | Less than 1 hour |  |  | **0.0982** |
| 1-3 hours |  | 0.49 | 0.21, 1.14 |  |
| >3 hours |  | 0.42 | 0.19, 0.93 |  |
|  |  |  |  |  |
| School type | State comprehensive school |  |  | 0.9831 |
| State grammar school |  | 1.01 | 0.47, 2.18 |  |
| Private school |  | 1.07 | 0.52, 2.19 |  |
|  |  |  |  |  |
| Time spent on live lessons per day | No time |  |  | 0.5729 |
| Some time |  | 1.18 | 0.67, 2.07 |  |
|  |  |  |  |  |
| Private academic tuition | No |  |  | 0.8263 |
| Yes |  | 0.90 | 0.34, 2.35 |  |
|  |  |  |  |  |
| Private music and drama tuition | No |  |  | 0.3727 |
| Yes |  | 0.75 | 0.40, 1.40 |  |
|  |  |  |  |  |
| Own PC for schoolwork | Yes |  |  | 0.2278 |
| No |  | 1.43 | 0.80, 2.56 |  |
|  |  |  |  |  |
| *Living situation* |  |  |  |  |
| House type | Other type of home |  |  | **0.1082** |
| House |  | 0.44 | 0.16, 1.20 |  |
|  |  |  |  |  |
| Who looked after young person | Mother and father |  |  | **0.1656** |
| Other |  | 1.54 | 0.84, 2.83 |  |
|  |  |  |  |  |
| Outdoor space | No access to outdoor space |  |  | 0.2902 |
| Private garden or outdoor space |  | 0.33 | 0.07, 1.65 |  |
| Shared garden or outdoor space |  | 0.58 | 0.08, 4.27 |  |
|  |  |  |  |  |
| Own bedroom in lockdown | No |  |  | 0.7821 |
| Yes |  | 0.89 | 0.39, 2.05 |  |
|  |  |  |  |  |
| *Other activities* |  |  |  |  |
| Time spent doing exercise per day | No time |  |  | 0.3927 |
| Up to 30 minutes |  | 0.66 | 0.24, 1.85 |  |
| 30 minutes - to 1 hour |  | 0.42 | 0.15, 1.15 |  |
| 1-3 hours |  | 0.45 | 0.16, 1.28 |  |
| >3 hours |  | 0.37 | 0.07, 1.98 |  |
|  |  |  |  |  |
| Number of times exercised per week | Less than once per week |  |  | 0.4933 |
| 1-3 times |  | 0.56 | 0.24, 1.28 |  |
| 4-5 times |  | 0.55 | 0.22, 1.35 |  |
| >5 times |  | 0.72 | 0.31, 1.67 |  |
|  |  |  |  |  |
| Time spent reading for fun per day | No time |  |  | 0.6692 |
| Up to 30 minutes |  | 0.88 | 0.46, 1.70 |  |
| 30 minutes - 1 hour |  | 0.59 | 0.25, 1.41 |  |
| >1 hours |  | 0.73 | 0.28, 1.92 |  |
|  |  |  |  |  |
| Time spent talking to friends on social media per day | No time |  |  | **0.1880** |
| Up to 30 minutes |  | 1.16 | 0.30, 4.44 |  |
| 30 minutes - 1 hour |  | 2.63 | 0.75, 9.26 |  |
| 1-3 hours |  | 1.72 | 0.48, 6.17 |  |
| >3 hours |  | 2.86 | 0.70, 11.23 |  |
|  |  |  |  |  |
| Time spent watching TV per day | No time |  |  | **0.1200** |
| 30 minutes - 1 hour |  | 0.54 | 0.18, 1.63 |  |
| 1-3 hours |  | 0.71 | 0.27, 1.87 |  |
| 4-6 hours |  | 1.34 | 0.46, 3.85 |  |
| >6 hours |  | 1.66 | 0.55, 5.03 |  |
|  |  |  |  |  |
| Time spent doing activities/skills per day | No time |  |  | **0.0450** |
| Up to 30 minutes |  | 0.20 | 0.07, 0.61 |  |
| 30 minutes - 1 hour |  | 0.56 | 0.24, 1.30 |  |
| 1-3 hours |  | 0.39 | 0.16, 0.96 |  |
| >3 hours |  | 0.28 | 0.06, 1.39 |  |
|  |  |  |  |  |
| Time spent playing games per day | No time |  |  | 0.4029 |
| Up to 30 minutes |  | 0.85 | 0.38, 1.89 |  |
| 30 minutes - 1 hour |  | 0.42 | 0.16, 1.12 |  |
| 1-3 hours |  | 0.68 | 0.29, 1.61 |  |
| >3 hours |  | 1.08 | 0.46, 2.52 |  |
|  |  |  |  |  |
| Time spent relaxing with household per day | No time |  |  | **0.0046** |
| Up to 30 minutes |  | 0.39 | 0.16, 0.93 |  |
| 30 minutes - 1 hour |  | 0.21 | 0.08, 0.53 |  |
| 1-3 hours |  | 0.18 | 0.07, 0.48 |  |
| >3 hours |  | 0.29 | 0.08, 1.02 |  |

^a^ Odds ratios and their 95% confidence intervals portray the direction and nature of the association with the capability-wellbeing outcome.
^b^ Univariable Wald tests: variables with p-values <0.2 were taken forward to within-group modelling stages.

*Supplementary Table 6 - Univariable logistic regression analyses for the capability to have relationships with family I don’t live with (odds ratios: ‘worse or much worse capability’ coded as one; ‘no change, more or much more capability’ coded as zero)*

| Explanatory variable | Reference category | Odds ratio^a^ | 95% confidence interval^a^ | p*-*value from univariable model^b^ |
| --- | --- | --- | --- | --- |
| *Sociodemographic characteristics* |  |  |  |  |
| IMD Quintile (categorical) | IMD 1 |  |  | **0.1910** |
| IMD 2 |  | 1.37 | 0.67, 2.78 |  |
| IMD 3 |  | 0.89 | 0.46, 1.74 |  |
| IMD 4 |  | 0.92 | 0.48, 1.78 |  |
| IMD 5 |  | 1.47 | 0.80, 2.70 |  |
|  |  |  |  |  |
| IMD Quintile (continuous) |  | 1.07 | 0.94, 1.22 | 0.2819 |
|  |  |  |  |  |
| Age group (years) | 11-12 |  |  | 0.2351 |
| 13 |  | 1.08 | 0.67, 1.74 |  |
| 14 |  | 0.84 | 0.52, 1.36 |  |
| 15-16 |  | 0.62 | 0.36, 1.07 |  |
|  |  |  |  |  |
| Gender | Female |  |  | 0.4776 |
| Male |  | 1.14 | 0.80, 1.63 |  |
|  |  |  |  |  |
| Ethnicity | White British |  |  | 0.9434 |
| Asian |  | 0.76 | 0.37, 1.53 |  |
| Black |  | 0.88 | 0.33, 2.29 |  |
| Mixed |  | 1.23 | 0.56, 2.71 |  |
| White other |  | 0.98 | 0.42, 2.30 |  |
| Other ethnic group |  | 1.40 | 0.33, 5.95 |  |
|  |  |  |  |  |
| Own dishwasher | No |  |  | 0.9434 |
| Yes |  | 1.12 | 0.73, 1.71 |  |
|  |  |  |  |  |
| Own vehicle | 0-1 vehicle |  |  | 0.8317 |
| 2 or more vehicles |  | 1.04 | 0.71, 1.52 |  |
|  |  |  |  |  |
| *Schooling and learning* |  |  |  |  |
| Time spent on schoolwork set by parents | No time |  |  | **0.0993** |
| Some time |  | 1.39 | 0.94, 2.05 |  |
|  |  |  |  |  |
| Hours of schoolwork set by school | Less than 1 hour |  |  | 0.8659 |
| 1-3 hours |  | 1.10 | 0.57, 2.10 |  |
| >3 hours |  | 1.17 | 0.63, 2.17 |  |
|  |  |  |  |  |
| School type | State comprehensive school |  |  | 0.5514 |
| State grammar school |  | 0.81 | 0.49, 1.34 |  |
| Private school |  | 1.13 | 0.72, 1.77 |  |
|  |  |  |  |  |
| Time spent on live lessons per day | No time |  |  | 0.8562 |
| Some time |  | 1.03 | 0.72, 1.47 |  |
|  |  |  |  |  |
| Private academic tuition | No |  |  | 0.7524 |
| Yes |  | 1.10 | 0.62, 1.95 |  |
|  |  |  |  |  |
| Private music and drama tuition | No |  |  | 0.7996 |
| Yes |  | 1.05 | 0.72, 1.52 |  |
|  |  |  |  |  |
| Own PC for schoolwork | Yes |  |  | 0.6835 |
| No |  | 1.08 | 0.74, 1.59 |  |
|  |  |  |  |  |
| *Living situation* |  |  |  |  |
| House type | Other type of home |  |  | 0.2697 |
| House |  | 0.64 | 0.30, 1.41 |  |
|  |  |  |  |  |
| Who looked after young person in lockdown | Mother and father |  |  | 0.8590 |
| Other |  | 1.04 | 0.69, 1.57 |  |
|  |  |  |  |  |
| Outdoor space | No access to outdoor space |  |  | **0.0591** |
| Private garden or outdoor space |  | 0.20 | 0.05, 0.83 |  |
| Shared garden or outdoor space |  | 0.31 | 0.06, 1.59 |  |
|  |  |  |  |  |
| Own bedroom in lockdown | No |  |  | 0.2762 |
| Yes |  | 1.38 | 0.77, 2.45 |  |
|  |  |  |  |  |
| *Other activities* |  |  |  |  |
| Time spent doing exercise per day | No time |  |  | 0.2553 |
| Up to 30 minutes |  | 1.10 | 0.49, 2.50 |  |
| 30 minutes - 1 hour |  | 1.43 | 0.65, 3.11 |  |
| 1-3 hours |  | 0.86 | 0.38, 1.92 |  |
| >3 hours |  | 1.35 | 0.47, 3.86 |  |
|  |  |  |  |  |
| Number of times exercised per week | Less than once per week |  |  | 0.2666 |
| 1-3 times |  | 1.24 | 0.70, 2.22 |  |
| 4-5 times |  | 1.21 | 0.65, 2.24 |  |
| >5 times |  | 0.81 | 0.43, 1.50 |  |
|  |  |  |  |  |
| Time spent reading for fun per day | No time |  |  | 0.3282 |
| Up to 30 minutes |  | 0.73 | 0.47, 1.12 |  |
| 30 minutes - 1 hour |  | 0.87 | 0.54, 1.46 |  |
| >1 hours |  | 0.61 | 0.33, 1.14 |  |
|  |  |  |  |  |
| Time spent talking to friends on social media per day | No time |  |  | 0.5192 |
| Up to 30 minutes |  | 0.72 | 0.38, 1.37 |  |
| 30 minutes - 1 hour |  | 0.70 | 0.37, 1.33 |  |
| 1-3 hours |  | 0.74 | 0.39, 1.39 |  |
| >3 hours |  | 0.46 | 0.19, 1.08 |  |
|  |  |  |  |  |
| Time spent watching TV per day | No time |  |  | 0.4645 |
| 30 minutes - 1 hour |  | 1.71 | 0.85, 3.45 |  |
| 1-3 hours |  | 1.31 | 0.68, 2.54 |  |
| 4-6 hours |  | 1.08 | 0.49, 2.35 |  |
| >6 hours |  | 1.26 | 0.54, 2.91 |  |
|  |  |  |  |  |
| Time spent doing activities/skills per day | No time |  |  | 0.8478 |
| Up to 30 minutes |  | 0.95 | 0.47, 1.93 |  |
| 30 minutes - 1 hour |  | 0.77 | 0.39, 1.51 |  |
| 1-3 hours |  | 0.81 | 0.41, 1.61 |  |
| >3 hours |  | 1.00 | 0.39, 2.56 |  |
|  |  |  |  |  |
| Time spent playing games per day | No time |  |  | **0.0471** |
| Up to 30 minutes |  | 0.67 | 0.39, 1.14 |  |
| 30 minutes - 1 hour |  | 0.70 | 0.41, 1.20 |  |
| 1-3 hours |  | 0.65 | 0.38, 1.13 |  |
| >3 hours |  | 1.40 | 0.80, 2.45 |  |
|  |  |  |  |  |
| Time spent relaxing with household per day | No time |  |  | **0.0218** |
| Up to 30 minutes |  | 0.88 | 0.41, 1.86 |  |
| 30 minutes - 1 hour |  | 0.60 | 0.29, 1.27 |  |
| 1-3 hours |  | 1.35 | 0.65, 2.81 |  |
| >3 hours |  | 1.04 | 0.42, 2.58 |  |

^a^ Odds ratios and their 95% confidence intervals portray the direction and nature of the association with the capability-wellbeing outcome.
^b^ Univariable Wald tests; variables with p-values <0.2 were taken forward to within-group modelling stages.

*Supplementary Table 7 - Univariable logistic regression analyses for the capability to have relationships with friends (odds ratios: ‘worse or much worse capability’ coded as one; ‘no change, more or much more capability’ coded as zero)*

| Explanatory variable | Reference category | Odds ratio^a^ | 95% confidence interval^a^ | p*-*value from univariable model^b^ |
| --- | --- | --- | --- | --- |
| *Sociodemographic characteristics* |  |  |  |  |
| IMD Quintile (categorical) | IMD 1 |  |  | 0.4654 |
| IMD 2 |  | 1.18 | 0.60, 2.32 |  |
| IMD 3 |  | 1.48 | 0.91, 2.72 |  |
| IMD 4 |  | 1.24 | 0.68, 2.27 |  |
| IMD 5 |  | 1.60 | 0.91, 2.83 |  |
|  |  |  |  |  |
| IMD Quintile (continuous) |  | 1.10 | 0.97, 1.24 | **0.1243** |
|  |  |  |  |  |
| Age group (years) | 11-12 |  |  | 0.3446 |
| 13 |  | 1.42 | 0.90, 2.24 |  |
| 14 |  | 1.38 | 0.88, 2.16 |  |
| 15-16 |  | 1.40 | 0.87, 2.27 |  |
|  |  |  |  |  |
| Gender | Female |  |  | 0.9148 |
| Male |  | 0.98 | 0.70, 1.37 |  |
|  |  |  |  |  |
| Ethnicity | White British |  |  | **0.0418** |
| Asian |  | 0.88 | 0.47, 1.63 |  |
| Black |  | 0.30 | 0.10, 0.91 |  |
| Mixed |  | 0.61 | 0.27, 1.38 |  |
| White other |  | 1.98 | 0.90, 4.37 |  |
| Other ethnic group |  | 0.19 | 0.02, 1.59 |  |
|  |  |  |  |  |
| Own dishwasher | No |  |  | 0.7521 |
| Yes |  | 0.94 | 0.64, 1.39 |  |
|  |  |  |  |  |
| Own vehicle | 0-1 vehicle |  |  | 0.3172 |
| 2 or more vehicles |  | 0.84 | 0.59, 1.19 |  |
|  |  |  |  |  |
| *Schooling and learning* |  |  |  |  |
| Time spent on schoolwork set by parents | No time |  |  | 0.3255 |
| Some time |  | 0.83 | 0.57, 1.20 |  |
|  |  |  |  |  |
| Hours of schoolwork set by school | Less than 1 hour |  |  | 0.3707 |
| 1-3 hours |  | 0.85 | 0.47, 1.53 |  |
| >3 hours |  | 1.10 | 0.63, 1.93 |  |
|  |  |  |  |  |
| School type | State comprehensive school |  |  | 0.8579 |
| State grammar school |  | 1.10 | 0.70, 1.71 |  |
| Private school |  | 0.94 | 0.62, 1.44 |  |
|  |  |  |  |  |
| Time spent on live lessons per day | No time |  |  | 0.2310 |
| Some time |  | 0.82 | 0.58, 1.14 |  |
|  |  |  |  |  |
| Private academic tuition | No |  |  | 0.9515 |
| Yes |  | 0.98 | 0.57, 1.70 |  |
|  |  |  |  |  |
| Private music and drama tuition | No |  |  | **0.1717** |
| Yes |  | 1.27 | 0.90, 1.80 |  |
|  |  |  |  |  |
| Own PC for schoolwork | Yes |  |  | 0.8059 |
| No |  | 1.05 | 0.73, 1.49 |  |
|  |  |  |  |  |
| *Living situation* |  |  |  | 0.8419 |
| House type | Other type of home |  |  |  |
| House |  | 0.92 | 0.43, 1.99 |  |
|  |  |  |  |  |
| Who looked after young person in lockdown | Mother and father |  |  | 0.3730 |
| Other |  | 1.19 | 0.81, 1.74 |  |
|  |  |  |  |  |
| Outdoor space | No access to outdoor space |  |  | 0.3154 |
| Private garden or outdoor space |  | 0.34 | 0.08, 1.38 |  |
| Shared garden or outdoor space |  | 0.37 | 0.07, 1.92 |  |
|  |  |  |  |  |
| Own bedroom in lockdown | No |  |  | 0.2221 |
| Yes |  | 1.38 | 0.82, 2.34 |  |
|  |  |  |  |  |
| *Other activities* |  |  |  |  |
| Time spent doing exercise per day | No time |  |  | 0.4269 |
| Up to 30 minutes |  | 0.60 | 0.29, 1.24 |  |
| 30 minutes - 1 hour |  | 0.72 | 0.36, 1.45 |  |
| 1-3 hours |  | 0.64 | 0.31, 1.30 |  |
| >3 hours |  | 0.41 | 0.15, .12 |  |
|  |  |  |  |  |
| Number of times exercised per week | Less than once per week |  |  | 0.4363 |
| 1-3 times |  | 0.65 | 0.38, 1.11 |  |
| 4-5 times |  | 0.81 | 0.46, 1.43 |  |
| >5 times |  | 0.74 | 0.43, 1.29 |  |
|  |  |  |  |  |
| Time spent reading for fun per day | No time |  |  | 0.3156 |
| Up to 30 minutes |  | 0.71 | 0.47, 1.06 |  |
| 30 minutes - 1 hour |  | 0.86 | 0.54, 1.39 |  |
| >1 hours |  | 0.67 | 0.38, 1.18 |  |
|  |  |  |  |  |
| Time spent talking to friends on social media per day | No time |  |  | **0.0008** |
| Up to 30 minutes |  | 0.79 | 0.43, 1.46 |  |
| 30 minutes - 1 hour |  | 0.56 | 0.30, 1.03 |  |
| 1-3 hours |  | 0.38 | 0.21, 0.70 |  |
| >3 hours |  | 0.30 | 0.13, 0.66 |  |
|  |  |  |  |  |
| Time spent watching TV per day | No time |  |  | 0.6579 |
| 30 minutes - 1 hour |  | 0.87 | 0.47, 1.63 |  |
| 1-3 hours |  | 0.72 | 0.40, 1.28 |  |
| 4-6 hours |  | 0.65 | 0.33, 1.30 |  |
| >6 hours |  | 0.86 | 0.41, 1.81 |  |
|  |  |  |  |  |
| Time spent doing activities/skills per day | No time |  |  | **0.1428** |
| Up to 30 minutes |  | 0.81 | 0.42, 1.57 |  |
| 30 minutes - 1 hour |  | 0.62 | 0.33, 1.16 |  |
| 1-3 hours |  | 0.74 | 0.39, 1.41 |  |
| >3 hours |  | 0.32 | 0.12, 0.85 |  |
|  |  |  |  |  |
| Time spent playing games per day | No time |  |  | **0.0595** |
| Up to 30 minutes |  | 0.83 | 0.51, 1.36 |  |
| 30 minutes - 1 hour |  | 0.59 | 0.36, 0.98 |  |
| 1-3 hours |  | 0.87 | 0.53, 1.44 |  |
| >3 hours |  | 0.47 | 0.27, 0.84 |  |
|  |  |  |  |  |
| Time spent relaxing with household per day | No time |  |  | **0.1418** |
| Up to 30 minutes |  | 0.49 | 0.24, 0.99 |  |
| 30 minutes - 1 hour |  | 0.41 | 0.20, 0.82 |  |
| 1-3 hours |  | 0.46 | 0.22, 0.92 |  |
| >3 hours |  | 0.38 | 0.16, 0.92 |  |

^a^ Odds ratios and their 95% confidence intervals portray the direction and nature of the association with the capability-wellbeing outcome.
^b^ Univariable Wald tests; variables with p-values <0.2 were taken forward to within-group modelling stages.

*Supplementary Table 8 - Multivariable logistic regression analyses for the capability of feeling safe and at ease (odds ratios: ‘worse or much worse capability’ coded as one; ‘no change, more or much more capability’ coded as zero)*

| Model stage |  | Within-group | | | Within-group/Across-groups^b^ | | |
| --- | --- | --- | --- | --- | --- | --- | --- |
| *Group* Explanatory variable | Reference category | Odds ratio | 95% confidence interval | p*-*value | Odds ratio | 95% confidence interval | p*-*value |
| *Sociodemographic characteristics* |  |  |  |  |  |  |  |
| Gender | Female |  |  | **0.0407** |  |  | **0.0407^b^** |
| Male |  | 0.61 | 0.39, 0.98 |  | 0.61 | 0.39, 0.98 |  |
| Own vehicle | 0-1 vehicle |  |  | **0.0086** |  |  | **0.0086^b^** |
| 2 or more vehicles |  | 0.54 | 0.34, 0.86 |  | 0.54 | 0.34, 0.86 |  |
|  |  |  |  |  |  |  |  |
| *Living situation* |  |  |  |  |  |  |  |
| Who looked after young person in lockdown | Mother and father |  |  | 0.1278^a^ |  |  |  |
| Other |  | 1.46 | 0.90, 2.40 |  |  |  |  |
| Own bedroom in lockdown | No |  |  | 0.2027^a^ |  |  |  |
| Yes |  | 0.67 | 0.36, 1.24 |  |  |  |  |

^a^ Dropping each of these variables in turn led back to the two univariable models depicted in Supplementary Table 1; since both of those (univariable) Wald test p-values failed to reach the pre-specified threshold (<0.10) for the within-group modelling, neither were taken forward to the across-groups models.
^b^ Both of these adjusted Wald test p-values reached the pre-specified threshold (<0.05) for the across-group modelling, and hence this within-group model is the final across-groups model for this outcome as depicted in Table 2. [Note that here and elsewhere, blank cells such as for this model indicate that the variable was not included in the model. Also, p-values are given in bold when they pass the threshold for that stage of modelling (10% for within-group, 5% for across-groups), and hence p-values not in bold indicate the stage at which a given variable was dropped from further consideration.]

*Supplementary Table 9 - Multivariable logistic regression analyses for the capability of talking and support (odds ratios: ‘worse or much worse capability’ coded as one; ‘no change, more or much more capability’ coded as zero)*

| Model stage |  | Within-group | | | Within-group | | | Across-groups | | |
| --- | --- | --- | --- | --- | --- | --- | --- | --- | --- | --- |
| *Group* Explanatory variable | Reference category | Odds ratio | 95% confidence interval | p*-*value | Odds ratio | 95% confidence interval | p*-*value | Odds ratio | 95% confidence interval | p-value |
| *Sociodemographic characteristics* |  |  |  |  |  |  |  |  |  |  |
| Age group | 11-12 |  |  | **0.0615** |  |  | **0.0476** |  |  | 0.1079^d^ |
| 13 |  | 1.11 | 0.51, 2.41 |  | 1.06 | 0.49, 2.28 |  | 1.15 | 0.51, 2.58 |  |
| 14 |  | 1.83 | 0.92, 3.65 |  | 1.92 | 0.97, 3.79 |  | 1.78 | 0.85, 3.71 |  |
| 15-16 |  | 2.33 | 1.15, 4.71 |  | 2.28 | 1.13, 4.57 |  | 2.28 | 1.08, 4.79 |  |
| Gender | Female |  |  | 0.1995^a^ |  |  |  |  |  |  |
| Male |  | 0.71 | 0.42, 1.20 |  |  |  |  |  |  |  |
| Own vehicle | 0-1 vehicle |  |  | 0.1161^a^ |  |  |  |  |  |  |
| 2 or more vehicles |  | 1.58 | 0.89, 2.80 |  |  |  |  |  |  |  |
|  |  |  |  |  |  |  |  |  |  |  |
| *Schooling and learning* |  |  |  |  |  |  |  |  |  |  |
| Time spent on schoolwork set by parents | No time |  |  | 0.1102^b^ |  |  |  |  |  |  |
| Some time |  | 0.61 | 0.34, 1.12 |  |  |  |  |  |  |  |
|  |  |  |  |  |  |  |  |  |  |  |
| *Other activities* |  |  |  |  |  |  |  |  |  |  |
| Number of times exercised per week | Less than once per week |  |  | 0.1365^c^ |  |  |  |  |  |  |
| 1-3 times |  | 0.55 | 0.27, 1.13 |  |  |  |  |  |  |  |
| 4-5 times |  | 0.37 | 0.16, 0.86 |  |  |  |  |  |  |  |
| >5 times |  | 0.55 | 0.25, 1.20 |  |  |  |  |  |  |  |
| Time spent watching TV per day | No time |  |  | 0.1369 |  |  | **0.0794** |  |  | 0.1416^d^ |
| 30 minutes - 1 hour |  | 0.55 | 0.19, 1.60 |  | 0.53 | 0.19, 1.53 |  | 0.53 | 0.17, 1.65 |  |
| 1-3 hours |  | 1.23 | 0.50, 3.00 |  | 1.17 | 0.48, 2.84 |  | 1.16 | 0.45, 3.01 |  |
| 4-6 hours |  | 1.74 | 0.64, 4.76 |  | 1.74 | 0.64, 4.75 |  | 1.59 | 0.54, 4.66 |  |
| >6 hours |  | 1.58 | 0.53, 4.71 |  | 1.79 | 0.61, 5.22 |  | 1.79 | 0.58, 5.55 |  |
| Time spent relaxing with household per day | No time |  |  | **0.0451** |  |  | **0.0430** |  |  | 0.1445^d^ |
| Up to 30 minutes |  | 0.53 | 0.22, 1.26 |  | 0.52 | 0.22, 1.22 |  | 0.55 | 0.22, 1.37 |  |
| 30 minutes - 1 hour |  | 0.38 | 0.16, 0.93 |  | 0.38 | 0.16, 0.90 |  | 0.42 | 0.17, 1.06 |  |
| 1-3 hours |  | 0.27 | 0.22, 0.70 |  | 0.27 | 0.11, 0.68 |  | 0.33 | 0.13, 0.85 |  |
| >3 hours |  | 0.23 | 0.06, 0.84 |  | 0.24 | 0.07, 0.86 |  | 0.28 | 0.07, 1.03 |  |

^a^ Adjusting gender and own vehicle only for age group led to p-values of 0.2050 and 0.1117 respectively and hence neither variable was considered for across-groups modelling and the final within-group model for this group was the (univariable) association with age group. ^b^ The single variable from this group did not meet the pre-specified threshold of p<0.10 for further (across-groups) modelling.
^c^ Adjusting this variable only for time spent relaxing yielded a p-value of 0.1073 and hence this variable was not included in the final within-group model presented in this table, with both those two variables warranting inclusion in the across-groups modelling.
^d^ From this three-variable model and the models considering the three two-variable models involving these three variables, none had p-values below 0.05 (as well as the one presented in the within-group model including TV watching and relaxing with household, that for age and TV watching led to p-values of 0.0700 and 0.2058 respectively, and that for age and time spent relaxing with household to p-values of 0.0634 and 0.2770 respectively). Consideration of the three univariable models led to a clear indication that the final model for this outcome should be the univariable model for age group (as the only one with a p-value<0.05), as depicted in the second column of this table and in Table 3.

*Supplementary Table 10 - Multivariable logistic regression analyses for the capability to have fun (odds ratios: ‘worse or much worse capability’ coded as one; ‘no change, more or much more capability’ coded as zero)*

| Model stage |  | Within-group | | | Within-group | | |
| --- | --- | --- | --- | --- | --- | --- | --- |
| *Group* Explanatory variable | Reference category | Odds ratio | 95% confidence interval | p*-*value | Odds ratio | 95% confidence interval | p*-*value^a^ |
| *Sociodemographic characteristics* |  |  |  |  |  |  |  |
| IMD Quintile (continuous) |  | 1.21 | 1.05, 1.40 | **0.0075** | 1.21 | 1.06, 1.38 | **0.0047** |
| Ethnicity | White British |  |  | **0.0186** |  |  | **0.0198** |
| Asian |  | 0.53 | 0.28, 1.00 |  | 0.53 | 0.28, 1.00 |  |
| Black |  | 0.75 | 0.30, 1.87 |  | 0.75 | 0.31, 1.86 |  |
| Mixed |  | 0.89 | 0.41, 1.92 |  | 0.90 | 0.42, 1.95 |  |
| White other |  | 4.38 | 1.46, 13.10 |  | 4.30 | 1.44, 12.80 |  |
| Other ethnic group |  | 0.38 | 0.09, 1.66 |  | 0.38 | 0.09, 1.65 |  |
| Own dishwasher | No |  |  | 0.6474^b^ |  |  |  |
| Yes |  | 0.90 | 0.56, 1.43 |  |  |  |  |
| Own vehicle | 0-1 vehicle |  |  | 0.6514^b^ |  |  |  |
| 2 or more vehicles |  | 1.09 | 0.74, 1.62 |  |  |  |  |
|  |  |  |  |  |  |  |  |
| *Schooling and learning* |  |  |  |  |  |  |  |
| Time spent on schoolwork set by parents | No time |  |  | 0.1054 |  |  |  |
| Some time |  | 0.73 | 0.51, 1.07 |  |  |  |  |
| Own PC for schoolwork | Yes |  |  | 0.1529 |  |  |  |
| No |  | 1.31 | 0.90, 1.89 |  |  |  |  |
|  |  |  |  |  |  |  |  |
| *Living situation* |  |  |  |  |  |  |  |
| Own bedroom in lockdown | No |  |  | 0.1498^c^ |  |  |  |
| Yes |  | 1.44 | 0.88, 2.37 |  |  |  |  |
|  |  |  |  |  |  |  |  |
| *Other activities* |  |  |  |  |  |  |  |
| Number of times exercised per week | Less than once per week |  |  | 0.2308^d^ |  |  |  |
| 1-3 times |  | 0.92 | 0.51, 1.64 |  |  |  |  |
| 4-5 times |  | 1.51 | 0.80, 2.84 |  |  |  |  |
| >5 times |  | 1.12 | 0.61, 2.08 |  |  |  |  |
| Time spent reading for fun per day | No time |  |  | **0.0089** |  |  | **0.0062** |
| Up to 30 minutes |  | 0.70 | 0.44, 1.12 |  | 0.68 | 0.43, 1.07 |  |
| 30 minutes - 1 hour |  | 0.57 | 0.34, 0.98 |  | 0.57 | 0.34, 0.95 |  |
| >1 hours |  | 0.36 | 0.20, 0.66 |  | 0.36 | 0.20, 0.64 |  |
| Time spent watching TV per day | No time |  |  | **0.0525** |  |  | **0.0275** |
| 30 minutes - 1 hour |  | 2.10 | 1.07, 4.11 |  | 2.22 | 1.15, 4.31 |  |
| 1-3 hours |  | 2.61 | 1.39, 4.91 |  | 2.65 | 1.43, 4.94 |  |
| 4-6 hours |  | 2.07 | 0.98, 4.36 |  | 1.89 | 0.91, 3.93 |  |
| >6 hours |  | 1.78 | 0.77, 4.10 |  | 1.61 | 0.72, 3.60 |  |
| Time spent doing activities/skills per day | No time |  |  | 0.2734^d^ |  |  |  |
| Up to 30 minutes |  | 1.20 | 0.56, 2.55 |  |  |  |  |
| 30 minutes - 1 hour |  | 0.86 | 0.42, 1.76 |  |  |  |  |
| 1-3 hours |  | 0.81 | 0.39, 1.69 |  |  |  |  |
| >3 hours |  | 0.49 | 0.19, 1.31 |  |  |  |  |
| Time spent playing games per day | No time |  |  | **0.0013** |  |  | **0.0015** |
| Up to 30 minutes |  | 0.53 | 0.31, 0.92 |  | 0.50 | 0.29, 0.87 |  |
| 30 minutes - 1 hour |  | 0.38 | 0.22, 0.67 |  | 0.39 | 0.23, 0.67 |  |
| 1-3 hours |  | 0.50 | 0.28, 0.88 |  | 0.50 | 0.29, 0.87 |  |
| >3 hours |  | 0.30 | 0.16, 0.56 |  | 0.31 | 0.17, 0.57 |  |

^a^ All five of the variables that remained from within-group modelling met the (more stringent) criterion of p<0.05 in the across-groups models and were therefore included in the final model for this outcome; the across-groups model is therefore not covered here since it is presented in full in Table 4.
^b^ Either together or separately, neither of these two variables warranted further consideration in the multivariable models.
^c^ For neither of these two final within-group models was the threshold for inclusion in the across-groups modelling met and hence neither of these variables were considered further for this outcome.
^d^ Either together or separately, neither of these two variables warranted further consideration in the multivariable models (adjusting only for the three variables retained, the p-values for number of times exercised and time spent on activities/skills were 0.2847 and 0.3760 respectively).

*Supplementary Table 11 - Multivariable logistic regression analyses for the capability of achieving the things that are important to me (odds ratios: ‘worse or much worse capability’ coded as one; ‘no change, more or much more capability’ coded as zero)*

| Model stage |  | Within-group | | | Across-groups | | | Across-groups | | |
| --- | --- | --- | --- | --- | --- | --- | --- | --- | --- | --- |
| *Group* Explanatory variable | Reference category | Odds ratio | 95% confidence interval | p*-*value | Odds ratio | 95% confidence interval | p*-*value | Odds ratio | 95% confidence interval | p*-*value |
| *Sociodemographic characteristics* |  |  |  |  |  |  |  |  |  |  |
| IMD Quintile (continuous) |  | 1.14 | 1.01, 1.28 | **0.0355**^a^ |  |  |  |  |  |  |
| Gender | Female |  |  | **0.0234** |  |  | **0.0233** |  |  | 0.0885^c^ |
| Male |  | 0.68 | 0.49, 0.95 |  | 0.68 | 0.49, 0.95 |  | 0.68 | 0.44, 1.06 |  |
|  |  |  |  |  |  |  |  |  |  |  |
| *Schooling and learning* |  |  |  |  |  |  |  |  |  |  |
| Time spent on schoolwork set by parents | No time |  |  | **0.0617**^a^ |  |  |  |  |  |  |
| Some time |  | 0.70 | 0.49, 1.02 |  |  |  |  |  |  |  |
|  |  |  |  |  |  |  |  |  |  |  |
| *Living situation* |  |  |  |  |  |  |  |  |  |  |
| House type | Other type of home |  |  | **0.0544** |  |  | **0.0379** |  |  |  |
| House |  | 2.18 | 0.98, 4.83 |  | 2.32 | 1.05, 5.13 |  |  |  |  |
| Who looked after young person in lockdown | Mother and father |  |  | **0.0885**^a^ |  |  |  |  |  |  |
| Other |  | 0.72 | 0.49, 1.05 |  |  |  |  |  |  |  |
|  |  |  |  |  |  |  |  |  |  |  |
| *Other activities* |  |  |  |  |  |  |  |  |  |  |
| Time spent doing exercise per day | No time |  |  | 0.7207^b^ |  |  |  |  |  |  |
| Up to 30 minutes |  | 0.87 | 0.35, 2.15 |  |  |  |  |  |  |  |
| 30 minutes - 1 hour |  | 0.90 | 0.34, 2.33 |  |  |  |  |  |  |  |
| 1-3 hours |  | 0.65 | 0.24, 1.77 |  |  |  |  |  |  |  |
| >3 hours |  | 0.82 | 0.23, 2.90 |  |  |  |  |  |  |  |
| Number of times exercised per week | Less than once per week |  |  | 0.3989^b^ |  |  |  |  |  |  |
| 1-3 times |  | 1.13 | 0.57, 2.24 |  |  |  |  |  |  |  |
| 4-5 times |  | 1.48 | 0.69, 3.17 |  |  |  |  |  |  |  |
| >5 times |  | 0.95 | 0.44, 2.08 |  |  |  |  |  |  |  |
| Time spent reading for fun per day | No time |  |  | **0.0006**^b^ |  |  |  |  |  | **0.0006**^c^ |
| Up to 30 minutes |  | 0.66 | 0.52, 1.05 |  |  |  |  | 0.70 | 0.44, 1.12 |  |
| 30 minutes - 1 hour |  | 0.71 | 0.41, 1.22 |  |  |  |  | 0.67 | 0.39, 1.16 |  |
| >1 hours |  | 0.26 | 0.14, 0.49 |  |  |  |  | 0.25 | 0.13, 0.49 |  |
| Time spent watching TV per day | No time |  |  | **0.0019**^b^ |  |  |  |  |  | **0.0054**^c^ |
| 30 minutes - 1 hour |  | 2.98 | 1.45, 6.13 |  |  |  |  | 2.96 | 1.43, 6.11 |  |
| 1-3 hours |  | 3.52 | 1.78, 6.97 |  |  |  |  | 3.33 | 1.67, 6.65 |  |
| 4-6 hours |  | 3.16 | 1.42, 7.02 |  |  |  |  | 3.11 | 1.38, 7.01 |  |
| >6 hours |  | 6.15 | 2.42, 15.6 |  |  |  |  | 5.40 | 2.10, 13.9 |  |
| Time spent doing activities/skills per day | No time |  |  | **0.0069**^b^ |  |  |  |  |  | **0.0047**^c^ |
| Up to 30 minutes |  | 1.21 | 0.56, 2.62 |  |  |  |  | 1.14 | 0.52, 2.51 |  |
| 30 minutes - 1 hour |  | 0.59 | 0.28, 1.24 |  |  |  |  | 0.55 | 0.26, 1.16 |  |
| 1-3 hours |  | 0.73 | 0.35, 1.54 |  |  |  |  | 0.70 | 0.33, 1.50 |  |
| >3 hours |  | 0.28 | 0.10, 0.79 |  |  |  |  | 0.25 | 0.09, 0.73 |  |
| Time spent playing games per day | No time |  |  | **0.0043**^b^ |  |  |  |  |  | **0.0466**^c^ |
| Up to 30 minutes |  | 0.63 | 0.36, 1.10 |  |  |  |  | 0.72 | 0.41, 1.27 |  |
| 30 minutes - 1 hour |  | 0.43 | 0.25, 0.74 |  |  |  |  | 0.47 | 0.27, 0.84 |  |
| 1-3 hours |  | 0.69 | 0.39, 1.22 |  |  |  |  | 0.85 | 0.45, 1.61 |  |
| >3 hours |  | 0.34 | 0.18, 0.64 |  |  |  |  | 0.46 | 0.23, 0.95 |  |
| Time spent relaxing with household per day | No time |  |  | **0.0842**^b^ |  |  |  |  |  | 0.0945^c^ |
| Up to 30 minutes |  | 0.80 | 0.36, 1.75 |  |  |  |  | 0.81 | 0.36, 1.79 |  |
| 30 minutes - 1 hour |  | 0.98 | 0.45, 2.13 |  |  |  |  | 0.95 | 0.43, 2.08 |  |
| 1-3 hours |  | 0.76 | 0.35, 1.66 |  |  |  |  | 0.76 | 0.35, 1.69 |  |
| >3 hours |  | 0.34 | 0.13, 0.89 |  |  |  |  | 0.33 | 0.12, 0.89 |  |

^a^ The p-value for hours of schoolwork set by parents was attenuated to 0.1184 after adjusting for IMD Quintile and gender (which themselves still had p-values <0.05) and hence the Schooling and learning group was not taken further in these analyses. Combining the first and the third group led to the p-values for IMD Quintile and who looked after young person in lockdown being similarly attenuated and so these variables were also dropped from further consideration.
^b^ Either with each other or not, neither of these two exercise variables warranted inclusion in the final within-groups model for this group, the results of which are given in this set of columns for the other five variables in this group.
^c^ Dropping the variable relating to relaxing with household led to p-values for the two marginal associations in this model (relating to gender and hours spent playing games) that were similar to each other at just over 0.05; since these appeared to be confounding each other the one with the smaller p-value in that model (gender) was retained for inclusion in the final model for this outcome presented in Table 5).

*Supplementary Table 12 - Multivariable logistic regression analyses for the capability to have relationships with the people I live with (odds ratios: ‘worse or much worse capability’ coded as one; ‘no change, more or much more capability’ coded as zero)*

| Model stage |  | Within-group | | | Within-group | | | Across-groups | | |
| --- | --- | --- | --- | --- | --- | --- | --- | --- | --- | --- |
| *Group* Explanatory variable | Reference category | Odds ratio | 95% confidence interval | p*-*value | Odds ratio | 95% confidence interval | p*-*value | Odds ratio | 95% confidence interval | p*-*value |
| *Sociodemographic characteristics* |  |  |  |  |  |  |  |  |  |  |
| IMD Quintile (continuous) |  | 0.79 | 0.62, 0.99 | **0.0452** | 0.76 | 0.62, 0.94 | **0.0104** | 0.74 | 0.58, 0.94 | **0.0157** |
| Age group | 11-12 |  |  | **0.0452** |  |  | **0.0401** |  |  | 0.6747 ^c^ |
| 13 |  | 1.41 | 0.55, 3.60 |  | 1.41 | 0.56, 3.60 |  | 1.36 | 0.50, 3.68 |  |
| 14 |  | 1.87 | 0.77, 4.52 |  | 1.85 | 0.76, 4.47 |  | 1.36 | 0.52, 3.56 |  |
| 15-16 |  | 3.19 | 1.36, 7.49 |  | 3.24 | 1.38, 7.59 |  | 1.82 | 0.70, 4.70 |  |
| Own dishwasher | No |  |  | 0.8583^a^ |  |  |  |  |  |  |
| Yes |  | 0.93 | 0.45, 1.94 |  |  |  |  |  |  |  |
| Own vehicle | 0-1 vehicle |  |  | 0.5066^a^ |  |  |  |  |  |  |
| 2 or more vehicles |  | 0.80 | 0.41, 1.55 |  |  |  |  |  |  |  |
|  |  |  |  |  |  |  |  |  |  |  |
| *Schooling and learning* |  |  |  |  |  |  |  |  |  |  |
| Hours of schoolwork set by school | Less than 1 hour |  |  | **0.0982** |  |  | **0.0982** |  |  | 0.3079^c^ |
| 1-3 hours |  | 0.49 | 0.21, 1.14 |  | 0.49 | 0.21, 1.14 |  | 0.47 | 0.17, 1.26 |  |
| >3 hours |  | 0.42 | 0.19, 0.93 |  | 0.42 | 0.19, 0.93 |  | 0.68 | 0.27, 1.72 |  |
|  |  |  |  |  |  |  |  |  |  |  |
| *Living situation* |  |  |  |  |  |  |  |  |  |  |
| House type | Other type of home |  |  | 0.1412 |  |  | 0.1082^b^ |  |  |  |
| House |  | 0.47 | 0.17, 1.29 |  | 0.44 | 0.16, 1.20 |  |  |  |  |
| Who looked after young person in lockdown | Mother and father |  |  | 0.2097 |  |  |  |  |  |  |
| Other |  | 1.48 | 0.80, 2.73 |  |  |  |  |  |  |  |
|  |  |  |  |  |  |  |  |  |  |  |
| *Other activities* |  |  |  |  |  |  |  |  |  |  |
| Time spent talking to friends on social media per day | No time |  |  | **0.1001** |  |  | **0.0723** |  |  | 0.1408 |
| Up to 30 minutes |  | 1.12 | 0.27, 4.55 |  | 1.18 | 0.30, 4.73 |  | 0.63 | 0.14, 2.73 |  |
| 30 minutes - 1 hour |  | 3.27 | 0.86, 12.47 |  | 3.26 | 0.87, 12.21 |  | 1.93 | 0.48, 7.83 |  |
| 1-3 hours |  | 2.40 | 0.61, 9.42 |  | 2.47 | 0.65, 9.44 |  | 1.47 | 0.36, 6.00 |  |
| >3 hours |  | 3.71 | 0.80, 17.26 |  | 4.31 | 0.96, 19.27 |  | 2.85 | 0.59, 13.72 |  |
| Time spent watching TV per day | No time |  |  | 0.2493 |  |  |  |  |  |  |
| 30 minutes - 1 hour |  | 0.52 | 0.16, 1.72 |  |  |  |  |  |  |  |
| 1-3 hours |  | 0.54 | 0.18, 1.56 |  |  |  |  |  |  |  |
| 4-6 hours |  | 1.20 | 0.37, 3.91 |  |  |  |  |  |  |  |
| >6 hours |  | 1.05 | 0.28, 3.87 |  |  |  |  |  |  |  |
| Time spent doing activities/skills per day | No time |  |  | **0.0516** |  |  | **0.0315** |  |  | **0.0399** |
| Up to 30 minutes |  | 0.19 | 0.06, 0.60 |  | 0.17 | 0.05, 0.53 |  | 0.17 | 0.05, 0.58 |  |
| 30 minutes - 1 hour |  | 0.50 | 0.20, 1.28 |  | 0.46 | 0.18, 1.15 |  | 0.54 | 0.20, 1.45 |  |
| 1-3 hours |  | 0.39 | 0.14, 1.03 |  | 0.36 | 0.14, 0.96 |  | 0.36 | 0.13, 1.03 |  |
| >3 hours |  | 0.21 | 0.04, 1.12 |  | 0.20 | 0.04, 1.09 |  | 0.19 | 0.03, 1.09 |  |
| Time spent relaxing with household per day | No time |  |  | **0.0006** |  |  | **0.0005** |  |  | **0.0037** |
| Up to 30 minutes |  | 0.34 | 0.13, 0.89 |  | 0.36 | 0.14, 0.92 |  | 0.42 | 0.15, 1.16 |  |
| 30 minutes - 1 hour |  | 0.14 | 0.05, 0.39 |  | 0.15 | 0.06, 0.41 |  | 0.18 | 0.06, 0.54 |  |
| 1-3 hours |  | 0.12 | 0.04, 0.36 |  | 0.12 | 0.04, 0.34 |  | 0.12 | 0.04, 0.40 |  |
| >3 hours |  | 0.17 | 0.04, 0.66 |  | 0.20 | 0.05, 0.77 |  | 0.20 | 0.05, 0.86 |  |

^a^ Either with each other or separately, neither of these two variables warranted inclusion in the final within-groups model for this group.
^b^ The final within-group model in this case was the univariable model for house type, and therefore no variable from this group warranted inclusion in the across-groups models for this outcome
^c^ Unlike that for time spent talking with friends on social media, either together or separately the variables of age group and hours of schoolwork set by the school had p-values above the threshold of 0.05 for this modelling stage; they were therefore dropped for the final model for this outcome presented in Table 6).

*Supplementary Table 13 - Multivariable logistic regression analyses for the capability to have relationships with family I don’t live with (odds ratios: ‘worse or much worse capability’ coded as one; ‘no change, more or much more capability’ coded as zero)*

| Model stage |  | Within-group | | | Across-groups | | |
| --- | --- | --- | --- | --- | --- | --- | --- |
| *Group* Explanatory variable | Reference category | Odds ratio | 95% confidence interval | p*-*value | Odds ratio | 95% confidence interval | p*-*value |
| *Sociodemographic characteristics* |  |  |  |  |  |  |  |
| IMD Quintile (categorical) | IMD 1 |  |  | 0.1910 |  |  |  |
| IMD 2 |  | 1.37 | 0.67, 2.78 |  |  |  |  |
| IMD 3 |  | 0.89 | 0.46, 1.74 |  |  |  |  |
| IMD 4 |  | 0.92 | 0.48, 1.78 |  |  |  |  |
| IMD 5 |  | 1.47 | 0.80, 2.70 |  |  |  |  |
|  |  |  |  |  |  |  |  |
| *Schooling and learning* |  |  |  |  |  |  |  |
| Time spent on schoolwork set by parents | No time |  |  | **0.0993** |  |  | 0.0876 ^a^ |
| Some time |  | 1.39 | 0.94, 2.05 |  | 1.43 | 0.95, 2.15 |  |
|  |  |  |  |  |  |  |  |
| *Living situation* |  |  |  |  |  |  |  |
| Outdoor space | No access to outdoor space |  |  | **0.0591** |  |  | 0.1135 ^a^ |
| Private garden or outdoor space |  | 0.20 | 0.05, 0.83 |  | 0.25 | 0.06, 1.02 |  |
| Shared garden or outdoor space |  | 0.31 | 0.06, 1.59 |  | 0.39 | 0.07, 2.26 |  |
|  |  |  |  |  |  |  |  |
| *Other activities* |  |  |  |  |  |  |  |
| Time spent playing games per day | No time |  |  | **0.0292** |  |  | 0.0539^b^ |
| Up to 30 minutes |  | 0.70 | 0.41, 1.22 |  | 0.65 | 0.38, 1.15 |  |
| 30 minutes - 1 hour |  | 0.67 | 0.39, 1.17 |  | 0.64 | 0.36, 1.11 |  |
| 1-3 hours |  | 0.62 | 0.35, 1.10 |  | 0.61 | 0.34, 1.08 |  |
| >3 hours |  | 1.41 | 0.80, 2.49 |  | 1.31 | 0.73, 2.36 |  |
| Time spent relaxing with household per day | No time |  |  | **0.0462** |  |  | 0.0629^b^ |
| Up to 30 minutes |  | 0.84 | 0.39, 1.81 |  | 0.90 | 0.42, 1.95 |  |
| 30 minutes - 1 hour |  | 0.61 | 0.29, 1.31 |  | 0.65 | 0.30, 1.40 |  |
| 1-3 hours |  | 1.36 | 0.64, 2.86 |  | 1.35 | 0.63, 2.86 |  |
| >3 hours |  | 0.87 | 0.34, 2.21 |  | 0.79 | 0.30, 2.04 |  |

^a^ Either with each other or separately, neither of these two variables warranted inclusion in the final model for this outcome presented in Table 7.
^b^ Once the other two variables were excluded, both of these two variables warranted inclusion in the final model for this outcome presented in Table 7.

*Supplementary Table 14 - Multivariable logistic regression analyses for the capability to have relationships with friends (odds ratios: ‘worse or much worse capability’ coded as one; ‘no change, more or much more capability’ coded as zero)*

| Model stage |  | Within-group | | | Within-group | | |
| --- | --- | --- | --- | --- | --- | --- | --- |
| *Group* Explanatory variable | Reference category | Odds ratio | 95% confidence interval | p*-*value | Odds ratio | 95% confidence interval | p*-*value |
| *Sociodemographic characteristics* |  |  |  |  |  |  |  |
| IMD Quintile (continuous) |  | 1.07 | 0.94, 1.22 | 0.3005 |  |  |  |
| Ethnicity | White British |  |  | **0.0586** |  |  | **0.0418** |
| Asian |  | 0.88 | 0.46, 1.67 |  | 0.88 | 0.47, 1.63 |  |
| Black |  | 0.35 | 0.11, 1.07 |  | 0.30 | 0.10, 0.91 |  |
| Mixed |  | 0.65 | 0.29, 1.47 |  | 0.61 | 0.27, 1.38 |  |
| White other |  | 2.11 | 0.95, 4.68 |  | 1.98 | 0.90, 4.37 |  |
| Other ethnic group |  | 0.20 | 0.02, 1.62 |  | 0.19 | 0.02, 1.59 |  |
|  |  |  |  |  |  |  |  |
| *Schooling and learning* |  |  |  |  |  |  |  |
| Private music and drama tuition | No |  |  | 0.1717^a^ |  |  |  |
| Yes |  | 1.27 | 0.90, 1.80 |  |  |  |  |
|  |  |  |  |  |  |  |  |
| *Other activities* |  |  |  |  |  |  |  |
| Time spent talking to friends on social media per day | No time |  |  | **0.0043** |  |  | **0.0008** |
| Up to 30 minutes |  | 0.92 | 0.48, 1.74 |  | 0.80 | 0.43, 1.49 |  |
| 30 minutes - 1 hour |  | 0.68 | 0.36, 1.29 |  | 0.55 | 0.29, 1.03 |  |
| 1-3 hours |  | 0.45 | 0.24, 0.87 |  | 0.39 | 0.21, 0.73 |  |
| >3 hours |  | 0.30 | 0.13, 0.73 |  | 0.27 | 0.11, 0.61 |  |
| Time spent doing activities/skills per day | No time |  |  | **0.0772** |  |  | 0.1517**^c^** |
| Up to 30 minutes |  | 0.71 | 0.34, 1.44 |  | 0.72 | 0.36, 1.44 |  |
| 30 minutes - 1 hour |  | 0.50 | 0.25, 1.01 |  | 0.58 | 0.30, 1.12 |  |
| 1-3 hours |  | 0.70 | 0.35, 1.40 |  | 0.72 | 0.37, 1.40 |  |
| >3 hours |  | 0.28 | 0.10, 0.80 |  | 0.31 | 0.11, 0.83 |  |
| Time spent playing games per day | No time |  |  | 0.2153**^b^** |  |  |  |
| Up to 30 minutes |  | 0.95 | 0.56, 1.61 |  |  |  |  |
| 30 minutes - 1 hour |  | 0.63 | 0.37, 1.07 |  |  |  |  |
| 1-3 hours |  | 0.94 | 0.55, 1.61 |  |  |  |  |
| >3 hours |  | 0.59 | 0.32, 1.08 |  |  |  |  |
| Time spent relaxing with household per day | No time |  |  | 0.2065**^b^** |  |  |  |
| Up to 30 minutes |  | 0.49 | 0.23, 1.03 |  |  |  |  |
| 30 minutes - 1 hour |  | 0.41 | 0.20, 0.87 |  |  |  |  |
| 1-3 hours |  | 0.53 | 0.25, 1.14 |  |  |  |  |
| >3 hours |  | 0.41 | 0.16, 1.07 |  |  |  |  |

^a^ This within-group model is essentially the univariable model for this one variable, which did not reach the threshold for consideration in the across-group modelling.
^b^ Either together or separately, neither of these two variables reached the threshold for further consideration in these models.
^c^ Since this variable did not meet the p<0.10 threshold it was not considered for across-groups modelling; hence the across-groups modelling involved just two variables (ethnicity and time talking to friends on social media) and both met the threshold for inclusion in the final model for this outcome as depicted in Table 8.
